# Lateral Gene Transfer Shapes Diversity of *Gardnerella* spp.

**DOI:** 10.3389/fcimb.2020.00293

**Published:** 2020-06-23

**Authors:** Lindsey L. Bohr, Tatum D. Mortimer, Caitlin S. Pepperell

**Affiliations:** ^1^Department of Medical Microbiology and Immunology, School of Medicine and Public Health, University of Wisconsin-Madison, Madison, WI, United States; ^2^Department of Immunology and Infectious Diseases, Harvard T.H. Chan School of Public Health, Boston, MA, United States; ^3^Department of Medicine, School of Medicine and Public Health, University of Wisconsin-Madison, Madison, WI, United States

**Keywords:** *Gardnerella* spp., recombination, evolution, bacterial vaginosis, lateral gene transfer

## Abstract

*Gardnerella* spp. are pathognomonic for bacterial vaginosis, which increases the risk of preterm birth and the transmission of sexually transmitted infections. *Gardnerella* spp. are genetically diverse, comprising what have recently been defined as distinct species with differing functional capacities. Disease associations with *Gardnerella* spp. are not straightforward: patients with BV are usually infected with multiple species, and *Gardnerella* spp. are also found in the vaginal microbiome of healthy women. Genome comparisons of *Gardnerella* spp. show evidence of lateral gene transfer (LGT), but patterns of LGT have not been characterized in detail. Here we sought to define the role of LGT in shaping the genetic structure of *Gardnerella* spp. We analyzed whole genome sequencing data for 106 *Gardnerella* strains and used these data for pan genome analysis and to characterize LGT in the core and accessory genomes, over recent and remote timescales. In our diverse sample of *Gardnerella* strains, we found that both the core and accessory genomes are clearly differentiated in accordance with newly defined species designations. We identified putative competence and pilus assembly genes across most species; we also found them to be differentiated between species. Competence machinery has diverged in parallel with the core genome, with selection against deleterious mutations as a predominant influence on their evolution. By contrast, the virulence factor vaginolysin, which encodes a toxin, appears to be readily exchanged among species. We identified five distinct prophage clusters in *Gardnerella* genomes, two of which appear to be exchanged between *Gardnerella* species. Differences among species are apparent in their patterns of LGT, including their exchange with diverse gene pools. Despite frequent LGT and co-localization in the same niche, our results show that *Gardnerella* spp. are clearly genetically differentiated and yet capable of exchanging specific genetic material. This likely reflects complex interactions within bacterial communities associated with the vaginal microbiome. Our results provide insight into how such interactions evolve and are maintained, allowing these multi-species communities to colonize and invade human tissues and adapt to antibiotics and other stressors.

## Introduction

*Gardnerella* spp. are Gram-variable, facultative anaerobes found in the vaginal microbiome of healthy women (Schellenberg et al., 2017). However, *Gardnerella* spp. are also associated with vaginal dysbiosis and bacterial vaginosis (BV), which is characterized by symptoms such as abnormal vaginal discharge, malodor, and pain (Hilbert et al., 2017). Additionally, BV can increase the risk of preterm birth and transmission of sexually transmitted diseases, including HIV (Hilbert et al., 2017).

*Gardnerella* spp. were previously considered a single species (i.e., *Gardnerella vaginalis*), but more recent research proposes that what was previously designated *G. vaginalis* in fact comprises several distinct species (Vaneechoutte et al., 2019). Biochemical tests (Piot et al., 1984) and phylogenetic methods (Ahmed et al., 2012; Cornejo et al., 2018) have delineated at least four distinct clades/species. While *Gardnerella* spp. are known to be associated with BV, clear consensus is lacking for the clades or combinations of clades that are most consequential for disease (Janulaitiene et al., 2017; Hill et al., 2019). Women with BV are often infected with strains from two or more clades (Hilbert et al., 2017). Recently, a study used average nucleotide identity and digital DNA-DNA hybridization to update the description of *G. vaginalis*, describe 3 new species, and 9 different genomospecies within “*Gardnerella* spp.” (Vaneechoutte et al., 2019). Researchers have tried to identify which groups of isolates are more pathogenic than others; however, this complex relationship remains to be fully defined (Harwich et al., 2010; Balashov et al., 2014; Janulaitiene et al., 2017; Hill et al., 2019). Our analyses build on previous work that used comparative genomics approaches to predict potential functional differentiation of this multi-species community (Cornejo et al., 2018). Here we investigate how these functional differences have evolved and are maintained in *Gardnerella* spp. populations.

We previously found evidence suggesting lateral gene transfer (LGT) is frequent among *Gardnerella* spp. (Devault et al., 2017). We and others found that LGT appeared to be structured by species, and that despite abundant intergenomic recombination, the species have maintained their genetic distinctiveness (Ahmed et al., 2012). Here we sought to characterize LGT in detail in a larger sample and to investigate whether patterns of recombination differ between species. The mechanism(s) of recombination in *Gardnerella* spp. are not known, but previous investigators found four predicted competence genes in a sample of three isolates (Yeoman et al., 2010). Additionally, prophage genes have been identified in *Gardnerella* spp. (Malki et al., 2016). As part of our investigation of LGT in *Gardnerella* spp. we further sought to identify and characterize genes with a potential role in shaping LGT, including competence genes, phage, and restriction modification systems, in a larger and more diverse sample of isolates. Elucidating how DNA is exchanged in bacterial populations helps to illuminate how clinically relevant traits, such as virulence and antibiotic resistance, evolve in these complex communities.

## Materials and Methods

### Data Set

We obtained whole genome sequencing reads or *de novo* assemblies (when reads were unavailable) for 97 isolates of *Gardnerella* spp. from NCBI. We additionally sequenced nine clinical *Gardnerella* spp. isolates. The accessions for the nine newly sequenced isolates are listed in Table S1. Accession numbers and available clinical data for all 106 isolates are listed in Table S2.

### Bacterial Growth and Isolation

Strains were streaked on human blood bilayer with Tween (HBT) agar (Bd Diagnostic Systems) and incubated at 37°C in 5% CO_2_ for 3–4 days. Growth was subcultured in 6 mls of brain heart infusion (BHI) broth (Teknova) + 10% fetal bovine serum (FBS) + 5% Fildes enrichment + 1 μg/mL amphotericin for 48 h at 37°C in 5% CO_2_.

### DNA Extraction

We used the gBac Mini gDNA Bacteria Kit (IBI, Lot No JM14117) for DNA extraction of 9 clinical isolates with the following modifications: the entire 6 ml culture was centrifuged for 5 min at 5,000 g to pellet cells and incubated with lysozyme for 1 h.

### Library Preparation and Sequencing

For the 9 new clinical isolates, libraries were prepared using a modified Nextera protocol as described by Baym et al. (2015) with a reconditioning PCR to minimize chimeras with fresh primers and polymerase for an additional 5 cycles and a bead based size selection (650 b). Libraries were sequenced on an Illumina HiSeq 2500 (paired-end, 150 bp).

### *De novo* Assembly and Annotation

We used the iMetAMOS (Koren et al., 2014) pipeline to compare *de novo* assemblies from SPAdes (Bankevich et al., 2012), MaSurCA (Zimin et al., 2013), and Velvet (Zerbino and Birney, 2008). KmerGenie (Chikhi and Medvedev, 2014) was used to select kmer sizes for assembly. Quality of reads and assemblies were assessed using FastQC (Andrews, 2010), QUAST (Gurevich et al., 2013), REAPR (Hunt et al., 2013), LAP (Ghodsi et al., 2013), ALE (Clark et al., 2013), FreeBayes (Garrison and Marth, 2012), and CGAL (Rahman and Pachter, 2013), and contamination was detected with Kraken (Wood and Salzberg, 2014). *De novo* assemblies were annotated with Prokka (Seemann, 2014).

### Core Genome Identification and Alignment

We used PIRATE (Bayliss et al., 2019) to identify the core and pan genomes for newly sequenced and publicly available *Gardnerella* spp. genomes (Table 1). Using PIRATE, we clustered orthologous gene families using an amino acid identity threshold ranging from 50 to 100% to obtain a clearer understanding of the breadth of diversity across the pangenomes of *Gardnerella* spp. We used the pangenome information from PIRATE and concatenated core genome alignments of single copy genes at 100% frequency for the entire dataset as well as individual species. In addition, we used Roary (Page et al., 2015) to identify core and pan genomes at an amino acid percent identity threshold of >75%. This allowed us to compare gene homologs at a consistent threshold across all genes.

**Table 1:** Number of genes identified in the pangenomes of 106 *Gardnerella* isolates.

**Program**		**Frequency of isolates**	**Number of genes**	**Amino acid % identity**
PIRATE	Core genes	99–100	608	50–100
	Soft core	95–99	195	50–100
	Total genes	0–100	4,653	50–100
Roary	Core genes	99–100	343	>75
	Soft core	95–99	275	>75
	Total genes	0–100	6,055	>75

*Amino acid percent identity used with Roary was inferred from the output of PIRATE, which uses a range of amino acid percent identity thresholds to calculate the pangenome*.

### Recombination Detection

To identify recombination events between the major clades/species of *Gardnerella* spp., we used FastGEAR (Mostowy et al., 2017) on a concatenated core genome aligned with MAFFT (Katoh and Standley, 2014). Briefly, FastGEAR uses a Hidden Markov Model approach to cluster isolates into lineages, detect ancestral and recent recombination, and measure the statistical strength of the recombination events. We used Gubbins (Croucher et al., 2015) to identify recombination events within the core genome alignments of clade 1 (*Gardnerella vaginalis*) and clade 2 (*Gardnerella piotii*). Briefly, Gubbins identifies recombination by using spatial scanning statistics to identify loci with elevated single nucleotide polymorphism (SNP) densities. To account for differences in sample size in *G. vaginalis* and *G. piotii*, we subsampled *G. vaginalis* to the size of *G. piotii* and used Gubbins to identify recombination in the subsampled dataset. We calculated the proportion of sites affected by recombination per isolate within *G. vaginalis* and *G. piotii* and compared the means using a Mann-Whitney-Wilcoxon test (Mann and Whitney, 1947).

### Codon Usage

We calculated codon adaptation index (CAI) for the core genes of *G. vaginalis* and *G. piotii* using the EMBOSS CUSP program (Rice, 2000). We compared the means of CAI values using a Student's *t*-test.

### Phylogenetic Network Inference

Using the concatenated core genome alignment constructed from PIRATE output, we inferred a phylogenetic network using SplitsTree 4 (Huson and Bryant, 2006) of all *Gardnerella* isolates, *G. vaginalis*, and *G. piotii* isolates.

### Maximum Likelihood Phylogenetic Inference

We performed maximum likelihood phylogenetic inference on the concatenated alignment of core genes using RAxML v 8.2.3 (Stamatakis, 2014) with the GTR model of nucleotide substitution and gamma distribution of rate variation. Twenty trees were estimated for the alignment, and the tree with the maximum likelihood was chosen. We performed bootstrapping using the autoMR convergence criteria.

### Competence Machinery and Vaginolysin Identification

We systematically identified competence genes, tad pilus assembly homologs, and vaginolysin using PIRATE and Roary gene homolog output, as well as manually using the Prokka (Seemann, 2014) annotations of the *Gardnerella* isolates. We included homologs of *cinA, recA, comEA, comEC, cpaB, cpaF, tadB, tadC, tadE*, and *tadG* in our selection analyses (Tomich et al., 2007; Yeoman et al., 2010). We aligned each gene with PRANK and constructed individual maximum likelihood gene trees using FastTree (Price et al., 2010). We used the aBSREL method implemented in Hyphy (Smith et al., 2015) to test for selection along branches in the phylogenies of competence genes. To investigate the effects of LGT and selection on the diversity of the competence machinery, vaginolysin, and the core genome, we compared these genomic regions in the two species that are well-sampled: *G. vaginalis* and *G. piotii*. We calculated dN/dS using the yn00 implementation (Yang and Nielsen, 2000) in PAML (Yang, 1997).

### Prophage Identification

We used ProphET (Reis-Cunha et al., 2019) to detect prophage in our collection of genomes. Briefly, ProphET performs a similarity search of annotated proteins from bacterial genomes against a database of known phage proteins to identify prophage within bacterial genomes. ProphET discards regions with a low density of phage-associated genes. Next, we blasted the known prophage regions against a custom nucleotide database of the *de novo* assembled contigs from all 106 *Gardnerella* spp. isolates to identify additional phage. To assess whether we missed prophage that were split between contigs, we blasted the known prophage against the custom database and filtered the results to identify hits found within 50 bp of a contig end and plotted the sequence length distribution of these hits. After identifying prophage regions, we calculated pairwise mash (Ondov et al., 2016) distances, which is based on shared k-mer (sequences of length k) content between prophage nucleotide sequences. Using these pairwise distances, we performed multidimensional scaling (MDS) to identify clusters of similar prophage. We also created a presence/absence matrix for each prophage cluster in our data set. To compare genetic content of each prophage cluster, we annotated the nucleotide sequences of all prophage using Prokka (Seemann, 2014), and cross referenced these results with the Clusters of Orthologous Groups (COG) database (Tatusov et al., 2000).

### CRISPR/*cas* Identification

We identified CRISPR/*cas* genes in genome annotations produced by Prokka. Additionally, we used the PIRATE output to identify homologous CRISPR/*cas* genes. To look for an association between the presence of CRISPR/*cas* and particular prophage cluster we used Fisher's Exact Test (Fisher, 1934) with Bonferroni correction (Bonferroni, 1935). We also performed a Mann-Whitney-Wilcoxon test (Mann and Whitney, 1947) to test for an association between the presence of CRISPR/*cas* and genome assembly length.

### Restriction Modification Identification

We used the Prokka annotations and PIRATE output to identify homologous clusters of genes associated with restriction modification (RM) systems. We created a presence/absence matrix for each RM associated gene in our data set.

### Pangenome Diversity Analyses

We calculated pangenome accumulation and rarefaction curves of *G. vaginalis* and *G. piotii* isolates. Additionally, we calculated the gene frequency of the accessory genomes of both species. Using Egglib (De Mita and Siol, 2012), we calculated average π per accessory gene within and between *G. vaginalis* and *G. piotii* isolates using a subset of genes found at intermediate frequencies (1–99%) in both species. We performed a Kruskal-Wallis test in R to determine differences in average gene π values by group (Kruskal and Wallis, 1952). We then performed pairwise Mann-Whitney-Wilcoxon tests in R with Bonferroni correction to identify which distribution pairs were significantly different (Bonferroni, 1935; Mann and Whitney, 1947).

## Results

### Pangenome and Phylogenetic Analysis of *Gardnerella* spp.

Pan genome analysis of 106 clinical isolates of *Gardnerella* spp. identified 4,653 genes in the pan-genome. Six hundred and eight of these formed the “strict core” genome (i.e., found in 100% of isolates) whereas an additional 195 genes were found in 95% of the sample (Table 1).

For initial categorization of genes in this diverse sample, we used an amino acid identity threshold of 50% or greater, implemented in PIRATE (Bayliss et al., 2019), to identify 4,653 gene families. We found an average of 75% amino acid percent identity among genes found in at least 95% of the isolates. Based on this finding, we performed additional pangenome analysis using Roary with an amino acid percent identity threshold of 75% to identify core and accessory genes (Table 1). At this more restrictive threshold, we found 6,055 total gene families, 343 of which were found in at least 99% of isolates.

**Figure 1:**
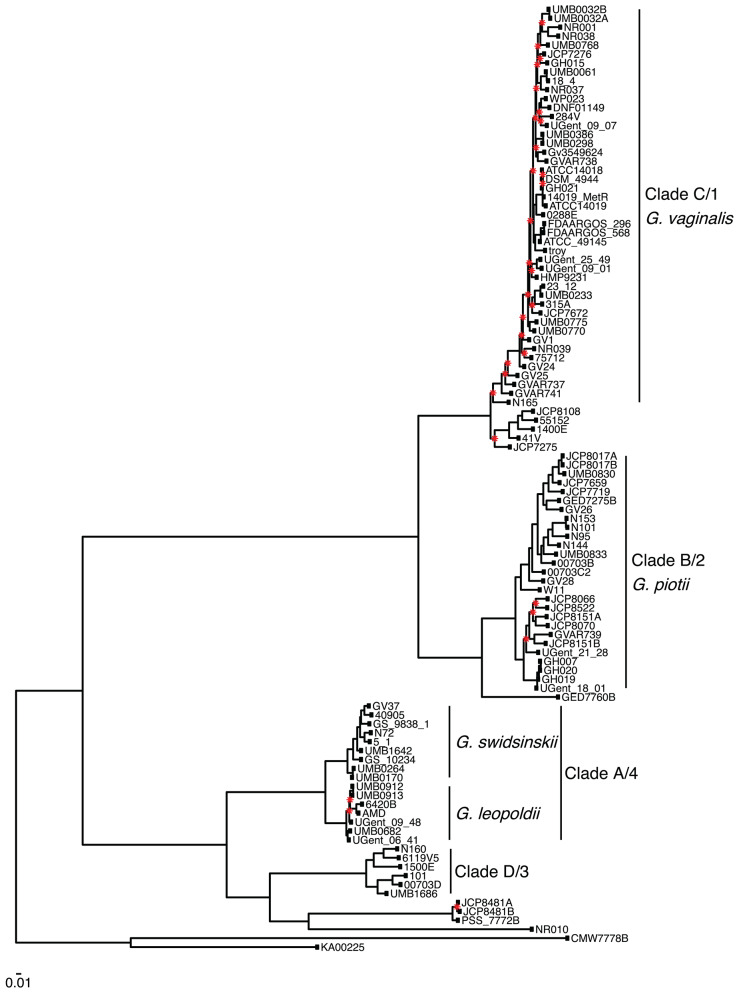
*Gardnerella* core genome maximum likelihood phylogeny supports distinct species/clade structure. We inferred a maximum likelihood phylogeny from a core genome alignment of 106 *Gardnerella* isolates. Species/clade labels reflect classification schemes from Ahmed et al. (2012) and Hill et al. (2019). Newly named species indicated (Vaneechoutte et al., 2019). The phylogeny is midpoint rooted, and nodes with bootstrap values lower than 70 shown in red. Branch lengths are scaled by the number of substitutions per site.

**Figure 2:**
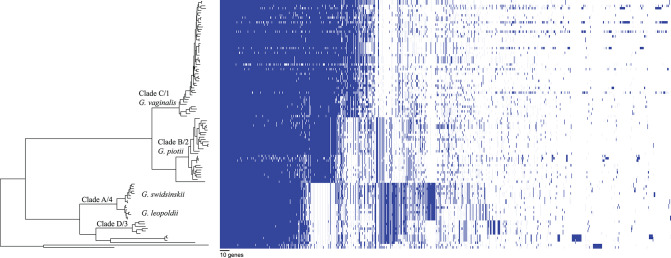
Accessory genome of *Gardnerella* is structured by species/clade. Gene homologs identified using Roary with 75% amino acid threshold are plotted (excluding singleton genes). Order of species in the phylogenetic tree (left) corresponds to species order in Figure 1. Accessory content differs among *Gardnerella* spp. species, which is consistent with barriers to LGT between species.

We found that *G. vaginalis, G. piotii, G. swidsinskii, G. leopoldii*, clade 3/D, and four unnamed additional clades were clearly differentiated in their core genomes, as shown by long branches separating them on a core genome phylogeny (Figure 1; Figure S1). The same pattern held in the accessory genome, where *Gardnerella* spp. could be clearly distinguished on the basis of accessory gene content (Figure 2).

### Lateral Gene Transfer in the *Gardnerella* Core Genome

**Figure 3:**
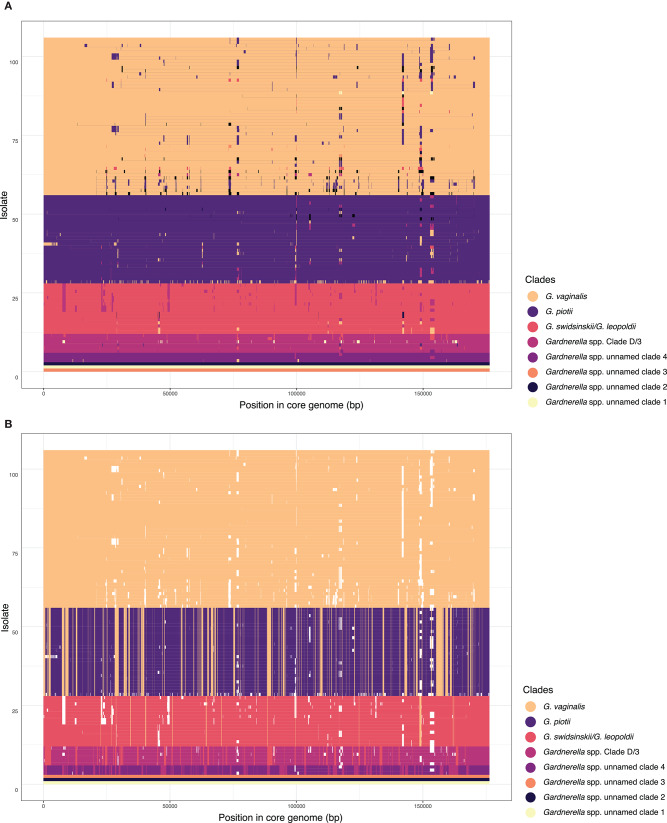
Recombination occurs most often between closely related species/clades. FastGEAR inference of recombination from a core genome alignment. Briefly, FastGEAR uses a Hidden Markov Model approach to cluster isolates into lineages, detect ancestral and recent recombination, and measure the statistical strength of the recombination events. FastGEAR identified 8 clades in the dataset, consistent with Figure 1 and published delineations (Ahmed et al., 2012; Schellenberg et al., 2017; Vaneechoutte et al., 2019). Isolates are ordered according to core genome phylogeny and colored according to each of 8 clades identified by FastGEAR. Each horizontal line refers to an isolate's core genome with colors representing the inferred origin of that region. The clades/species colors are labeled in the legend. **(A)** Recent recombinant tracts identified with FastGEAR. Overall there are few recombination events between clades/species, which appear to be more common between *G. vaginalis* and *G. piotii*, than other combinations. **(B)** Ancestral recombination shows a similar pattern of species structured LGT. White fragments correspond to recent recombination events (shown in **A**) and are masked when inferring ancestral recombination. Recent recombination inferred with a Bayesian factor (BF) > 1 and ancestral recombination with BF > 10 shown.

Our analyses of lateral gene transfer indicated that this species structure is maintained by barriers to recombination. We used FastGEAR to identify sub-groups within the sample and infer patterns of core genome recombination in the recent and remote past. FastGEAR analyses defined eight clades, consistent with previously published genetic classifications. Few recombination events were inferred between these eight clades (Figure 3A). Interestingly, between-clade recombination appeared to have occurred more commonly in the remote past (Figure 3B). More recombination events were inferred between closely related clades/species (i.e., between *G. vaginalis* and *G. piotii*) than distantly related clades/species (e.g., *G. vaginalis* and clade D/3).

### Mechanisms of LGT

**Figure 4:**
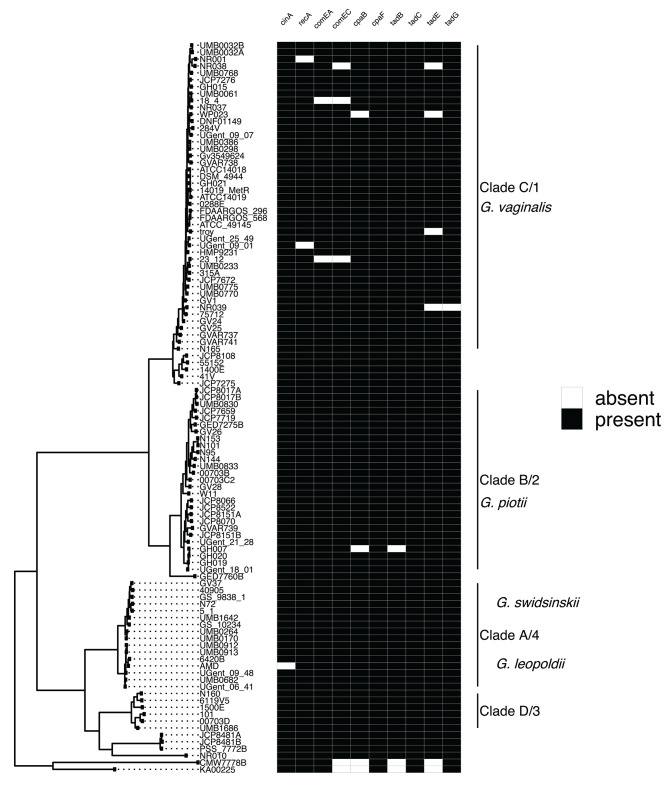
Competence gene homologs are ubiquitous across *Gardnerella*. Presence absence matrix of gene homologs likely to be related to competence **(right)** and maximum likelihood phylogenetic tree **(left)**. Most of the genes are found among all clades/species, indicating competence related machinery is conserved among *Gardnerella* spp. Gene homologs were identified using both Roary and PIRATE. Additionally, we looked in the annotations to identify homologs missed using Roary or PIRATE alone.

To identify potential mechanisms of LGT in *Gardnerella*, we first systematically searched for and identified a collection of competence related genes. A previous study identified four competence genes (*cinA, recA, comEA, comEC*) in a sample of three *Gardnerella* isolates (Yeoman et al., 2010). We expanded this finding to our collection of 106 *Gardnerella* isolates and identified 6 additional genes (*cpaB, cpaF, tadB, tadC, tadE, tadG*) involved in tad pilus assembly (Tomich et al., 2007). We found competence homologs to be encoded by most isolates (Figure 4). These genes were highly differentiated among species (e.g., Figure S2), mirroring patterns of diversity in the core genome. This suggests that competence genes are not commonly exchanged across species.

**Figure 5:**
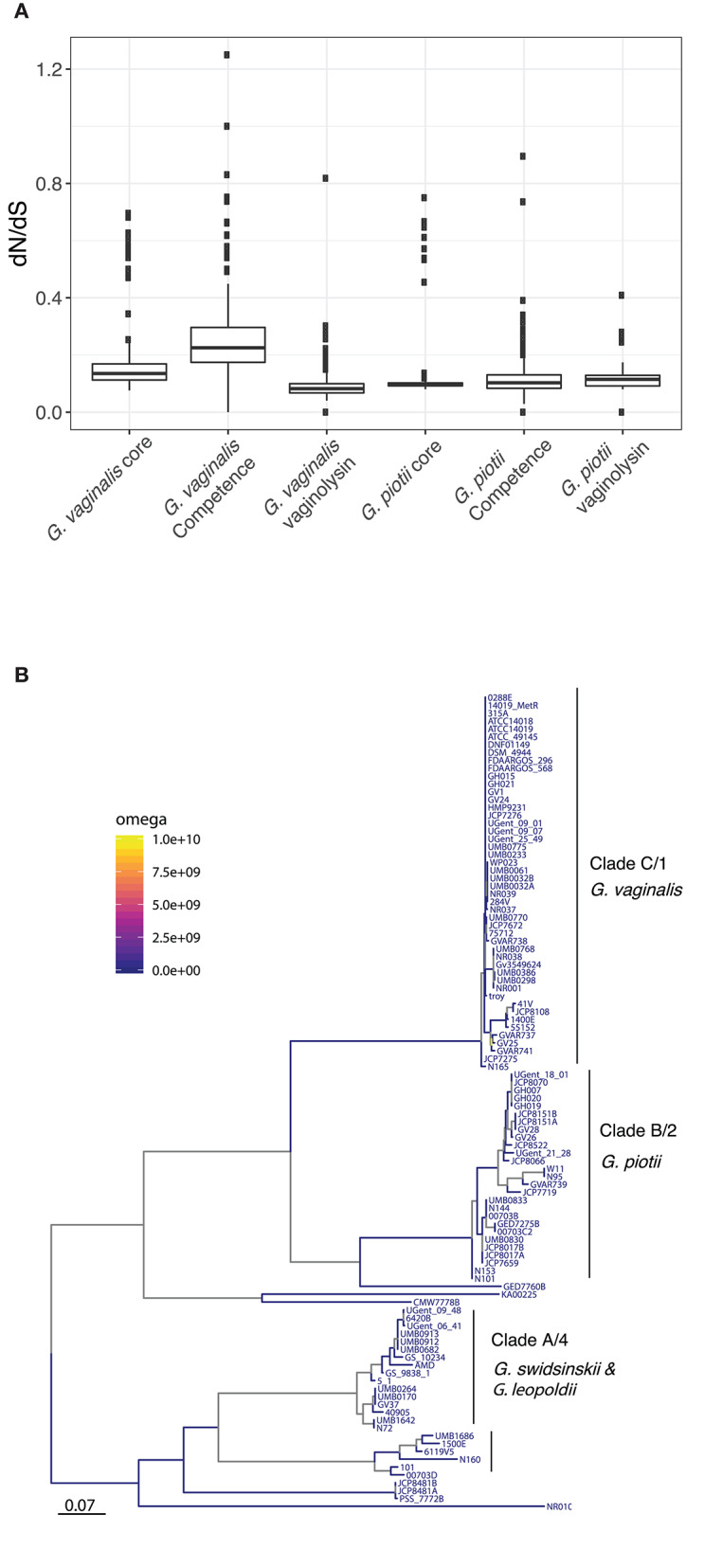
Purifying selection is the primary force shaping diversity in *Gardnerella* core, competence, and vaginolysin genes. **(A)** Diversity of core genomes, competence genes, and vaginolysin within *G. vaginalis* and *G. piotii*. Distributions shown are of dN/dS across core genomes, concatenations of competence genes, and vaginolyisn. The box spans the interquartile range, the median is represented by the middle line, and the whiskers extend to ±1.5 times the interquartile range. Data beyond the end of the whiskers are outlying points and plotted individually. All genes appear to be under purifying selection (dN/dS <1). **(B)** Proportion of sites under positive selection along branches of the *comEA* maximum likelihood phylogeny. Using the aBSREL test in HyPhy, we identified branches with significant evidence (*p* < 0.05) of selection. Branch specific omega values show little evidence of positive selection, suggesting purifying selection.

It is possible that patterns of diversity in competence genes reflect functional differentiation that could contribute to reproductive isolation among *Gardnerella* spp. To test this hypothesis, we performed selection analyses: in the event that divergence was driven by functional differentiation we expect to find evidence of positive selection reflected in excess non-synonymous (coding) variation separating species-specific versions of these genes. Pairwise values of dN/dS are consistent with purifying selection (i.e., selection against deleterious mutations) as the predominant influence on both core genes and competence homologs (Figure 5A). Estimates of branch specific omega values also indicate that the competence genes are generally evolving under purifying selection (Figure 5B; Figure S3). Thus, patterns of diversity in competence machinery do not suggest that their differentiation has been driven by selection for advantageous mutations/ functional differentiation.

### Patterns of Diversity at a Virulence Locus

Vaginolysin is a pore-forming toxin and acute virulence factor (Gelber et al., 2008) previously identified as a core gene with evidence of between-clade recombination in a sample of 17 isolates (Ahmed et al., 2012). Here we sought to further investigate evolution of this locus: genes that mediate host pathogen interactions are often characterized by high levels of diversity reflecting selection for advantageous mutations (Andrews and Gojobori, 2004; Kennemann et al., 2011; Osório et al., 2013) and we were interested in dynamics of this well-characterized virulence factor in *Gardnerella* spp. By contrast with Ahmed et al., we did not find vaginolysin to be part of the core genome in this larger sample of isolates. Using a >80% amino acid similarity threshold in the PIRATE gene homolog output, we found vaginolysin to be present in 83% of *Gardnerella* isolates. A presence/absence matrix for this gene implies that vaginolysin has been gained or lost a limited number of times during evolution of our sample (Figure S4). The vaginolysin gene tree is not consistent with the core phylogeny, indicating that while it is infrequently lost or gained, it appears to be readily exchanged between *Gardnerella* spp. (Figures S4, S5). Interestingly, we found that vaginolysin appears to be evolving under strong constraint, similar to the core genome and competence machinery in *G. vaginalis* (Figure 5A). Taken together, these results suggest that the vaginolysin toxin performs an important function in *Gardnerella* spp., but bacteria occasionally adapt to loss of this function.

### Prophage

**Figure 6:**
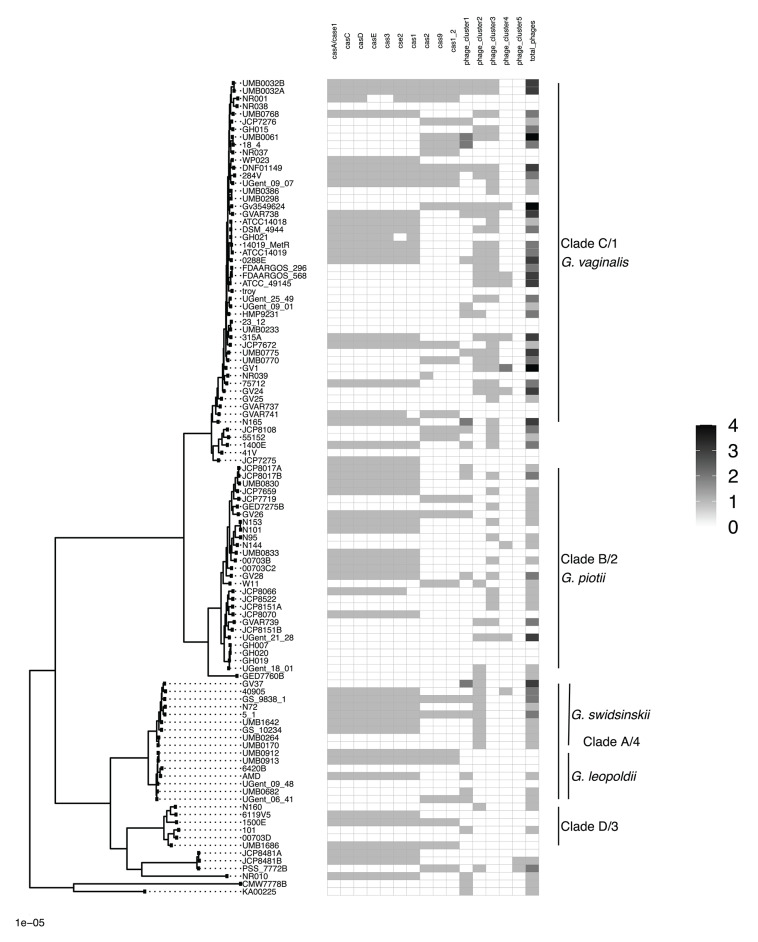
CRISPR/*cas* and prophage are not ubiquitous across *Gardnerella* clades/species. Presence absence matrix of CRISPR/*cas* genes and phage clusters. CRISPR/*cas* genes were identified using PIRATE output and Prokka genome annotations. We identified prophage regions using ProphET and calculated pairwise mash distances of the nucleotide sequences to define prophage clusters. Prophage clusters 1 and 2 are found across *Gardnerella* spp., while prophage clusters 3 and 4 are restricted to *G. vaginalis* and *G. piotii*, with the exception of one cluster 4 prophage found in *G. swidsinskii*. We identified prophage clusters in 70% of *Gardnerella* isolates.

**Figure 7:**
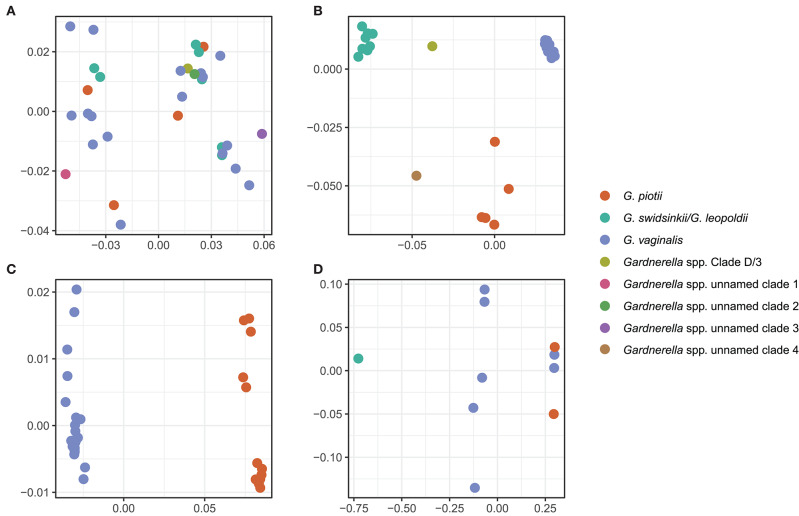
Prophage clusters have distinct clade/species restrictions and patterns of transfer between clades/species. We used multi-dimensional scaling (MDS) of pairwise mash distances of prophage nucleotide sequences identified using ProphET to visualize prophage clustering across clades/species. Prophage clusters 2 and 3 cluster based on the core genome species designations **(B,C)**, and prophage clusters 1 and 4 do not cluster according to core genome species designations **(A,D)**, suggesting that the prophage have been transferred across clades. We did not include an MDS plot of cluster 5, as it is found in only 2 isolates.

To further explore mechanisms of LGT, we used ProphET (Reis-Cunha et al., 2019) to identify 130 prophage within our set of 106 genomes. We found *Gardnerella* spp. to encode between zero and four prophage per genome (median 1). Using mash distances (Ondov et al., 2016), we identified 5 clusters of prophage, with at least one prophage found in 70% of *Gardnerella* isolates. Prophage clusters 1 and 2 are found across *Gardnerella* spp. (Figure 6), while prophage clusters 3 and 4 are restricted to *G. vaginalis* and *G. piotii*, with the exception of one cluster 4 prophage found in a *G. swidsinskii* isolate. Cluster 5 is the smallest group, found in 2 isolates from an unnamed Gardnerella spp. clade (Figure 6). Phages in clusters 2 and 3 form well-differentiated sub-clusters that correspond with host species designations (Figures 7B,C), whereas cluster 1 and 4 phages from different hosts co-mingle (Figures 7A,D). This suggests there are barriers to between-species transfer of phage clusters 2 and 3 whereas clusters 1 and 4 are readily transferred among diverse bacterial hosts. We found the genetic content of prophage to vary between clusters (Figure S6); however, the majority of the genes were uncharacterized hypothetical genes (82%). To assess if we missed prophage split over contigs, we blasted all prophage against a custom database of all *de novo* assembled contigs and filtered the results to find hits within 50 bp of the end of a contig. We plotted the sequence length distribution of these blast results and found the majority were very small in length, which suggests they are unlikely to be identified prophage (Figure S7).

### CRISPR/*cas*

CRISPR/*cas* protect bacterial genomes from foreign DNA, including phage, and thus could potentially play a role in maintaining genetic barriers among *Gardnerella* spp. Presence/absence matrix of CRISPR/*cas* loci in our sample is shown in Figure 6. Patterns of carriage were variable, with some strains carrying up to 10 distinct loci and others without any loci identified. Certain loci tended to co-occur. Loss of CRISPR/*cas* can lead to proliferation of mobile genetic elements (Hullahalli et al., 2018), in which case we might expect genomes lacking CRISPR/*cas* to accumulate mobile genetic elements and increase in size. To test this hypothesis, we compared the presence of CRISPR/*cas* to the total length of *de novo* assembled contigs. We found that CRISPR/*cas* is not associated with increased genome length (Mann-Whitney-Wilcoxon test, W = 1,058, *p* = 0.08) (Figure S8).

### Restriction Modification

Restriction modification (RM) systems can also protect bacterial genomes by cleaving foreign DNA (Tock and Dryden, 2005), and thus may shape barriers to lateral gene transfer in *Gardnerella* spp. To identify genes involved in restriction modification (RM) systems, we used PIRATE homologous gene family output and Prokka genome annotations. We found a wide diversity of RM genes found at varying frequencies across the *Gardnerella* spp. phylogeny, and *Gardnerella* spp. are not defined by the presence/absence of particular RM gene families (Figure S9).

### Codon Usage in *G. vaginalis* and *G. piotii*

Differences in codon usage (Tuller et al., 2011) are another potential mechanism driving differentiation and genetic isolation of *Gardnerella* species. To investigate this hypothesis, we focused on *G. vaginalis* and *G. piotii*, sister species in the *Gardnerella* phylogeny that are common and well-sampled (50 *G. vaginalis*, 28 *G. piotii*). We found codon usage to be similar in *G. vaginalis* and *G. piotii* isolates and thus it does not appear to be an explanation for the reproductive isolation of these species (*t*-test, *p* > 0.99) (Figure S10).

### Lateral Gene Transfer in Core Genomes of *G. vaginalis* and *G. piotii*

**Figure 8:**
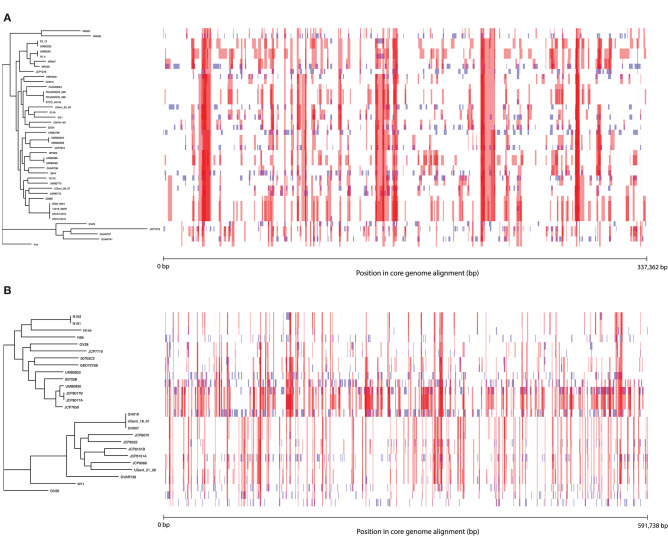
Within species recombination is frequent in *Gardnerella* core genomes. Recombination in the core genomes of *G. vaginalis* **(A)** and *G. piotii* **(B)**. Each row corresponds to the core genome of an isolate in the phylogenetic tree to the left. Blue segments represent laterally transferred fragments unique to an individual isolate. Red segments indicate laterally transferred fragments that are shared across multiple isolates. The proportion of the alignment affected by recombination (both red and blue fragments) is 0.94 for *G. vaginalis* and 0.92 for *G. piotii*.

**Figure 9:**
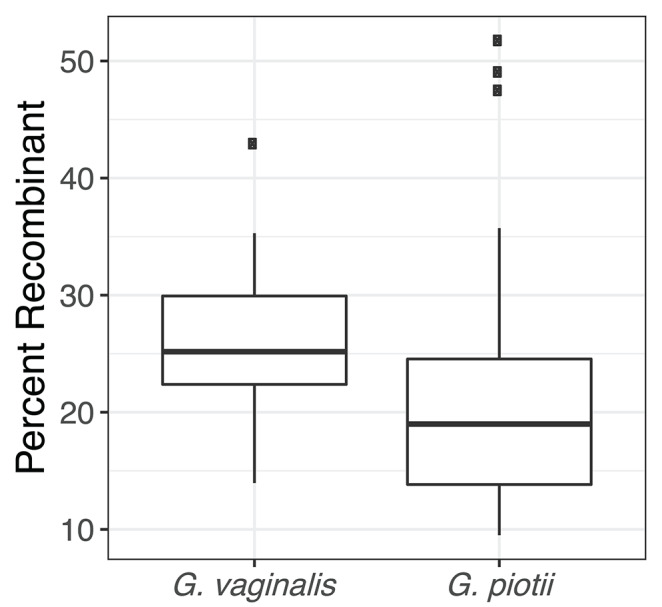
*G. vaginalis* core genomes are more recombinant than *G. piotii*. Boxplots show proportion of each isolate's core genome affected by recombination, as estimated with Gubbins. The box spans the interquartile range, the median is represented by the middle line, and the whiskers extend to ±1.5 times the interquartile range. Data beyond the end of the whiskers are outlying points and plotted individually. To account for differences in sample size of *G. vaginalis* and *G. piotii* isolates, we subsampled *G. vaginalis* isolates to the number of *G. piotii* isolates and used Gubbins to identify recombination in the subsampled dataset. The mean of affected core genomes in the subsampled *G. vaginalis* his higher than that of *G. piotii* (Mann-Whitney-Wilcoxon test, W = 473, *p* = 0.0138).

To further characterize patterns of recombination in *G. vaginalis* and *G. piotii*, we identified recombinant tracts in core genomes with Gubbins. LGT appears qualitatively more frequent in *G. vaginalis* than in *G. piotii* (Figure 8). The proportion of the total alignment affected by recombination is similar in the two species (0.94 for *G. vaginalis* and 0.92 for *G. piotii*), in an uncorrected analysis. To account for differences in sample size in *G. vaginalis* and *G. piotii* isolates, we sub-sampled the number of *G. vaginalis* isolates to the number of *G. piotii* isolates and used Gubbins to identify recombination in the subsampled dataset. The mean proportion of each isolate's core genome affected by recombination is greater for *G. vaginalis* (26.1%) than for *G. piotii* (23.1%) (Mann-Whitney-Wilcoxon test, W = 473, *p* = 0.0138) (Figure 9). The *G. vaginalis* network contains more reticulations than *G. piotii*, which also supports overall higher rates of recombination in *G. vaginalis* (Figure S11).

### Patterns of Lateral Gene Transfer in Accessory Genomes of *G. vaginalis* and *G. piotii*

**Figure 10:**
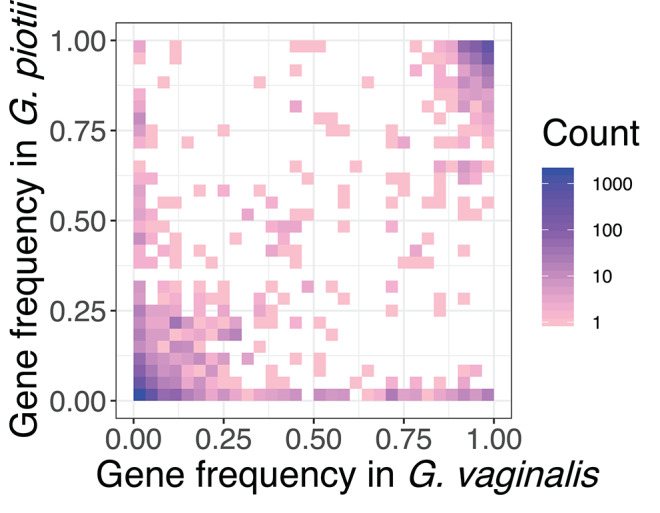
Accessory genes are not maintained at similar frequencies in *G. vaginalis* and *G. piotii*. Heat map of pangenome gene frequencies in *G. vaginalis* and *G. piotii*. Accessory genes are not maintained at similar frequencies in the two species suggesting that selection pressures for the shared accessory genes are not the same across species. For example, some genes are maintained at high frequencies in one species, but low in the other. Number of genes are colored on a log scale.

Inter-species barriers to gene exchange were evident in comparisons of the accessory genomes of *G. vaginalis* and *G. piotii*. Accessory gene frequencies *in G. vaginalis* and *G. piotii* isolates indicated that most accessory genes were unique to an individual species, with few genes found at similar frequencies across species (Figure 10). If accessory genes were freely exchanged between species, we would expect their frequencies to equilibrate.

**Figure 11:**
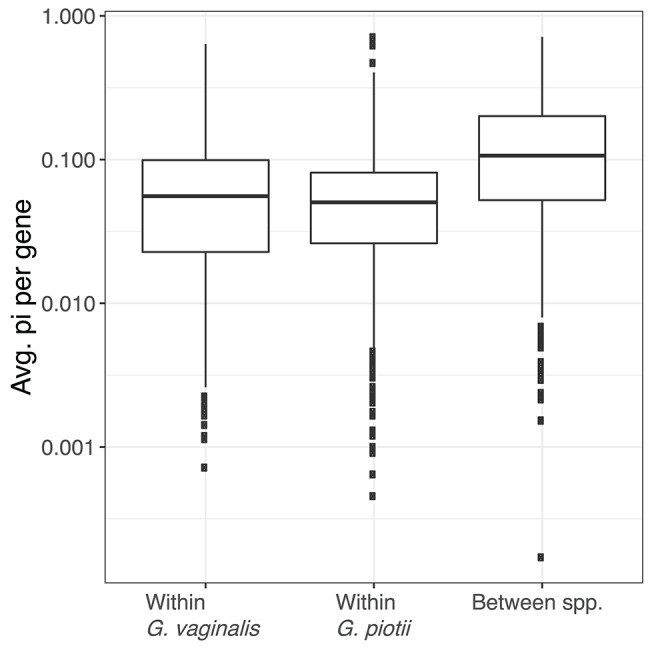
Accessory gene flow occurs more often within *Gardnerella* species than between species. The distributions of nucleotide diversity (π) per shared accessory gene within and between *G. vaginalis* and *G. piotii*, were calculated and log transformed. The box spans the interquartile range, the median is represented by the middle line, and the whiskers extend to ±1.5 times the interquartile range. Data beyond the end of the whiskers are outlying points and plotted individually. Average gene π values differ significantly by group (Kruskal-Wallis test, H = 214.7, *p* < 2.2e-16). The distributions of average gene π values of *G. vaginalis* (Mann-Whitney-Wilcoxon, W = 373,928, *p* < 3.322e-13) and *G. piotii* (Mann-Whitney-Wilcoxon, W = 399,762, *p* < 3.37e-13) are lower than between species. In species that regularly exchange accessory gene content, we might expect to see similar levels of diversity in shared accessory gene content. However, we observed lower diversity within species, consistent accessory gene flow occurring more often within *G. vaginalis* and *G. piotii* than between them.

To further characterize lateral gene transfer in the accessory genome, we quantified pairwise diversity (π) for accessory genes common to *G. vaginalis* and *G. piotii*. We performed a Kruskal-Wallis test and found there was a statistically significant difference between average gene π values by group (H = 214.7, *p* < 2.2e-16). We then performed pairwise Mann-Whitney-Wilcoxon tests with Bonferroni correction and found the distributions of average gene π values of *G. vaginalis* (W = 373,928, *p* < 3.32e-13) and *G. piotii* isolates (W = 399,762, *p* < 3.37e-13) were lower than between species (Figure 11). In species that regularly exchange accessory gene content, we expect to see similar levels of diversity in between- and within-species comparisons. However, we observed lower diversity within species, indicating that accessory gene variants are transferred more frequently within species than between them.

**Figure 12:**
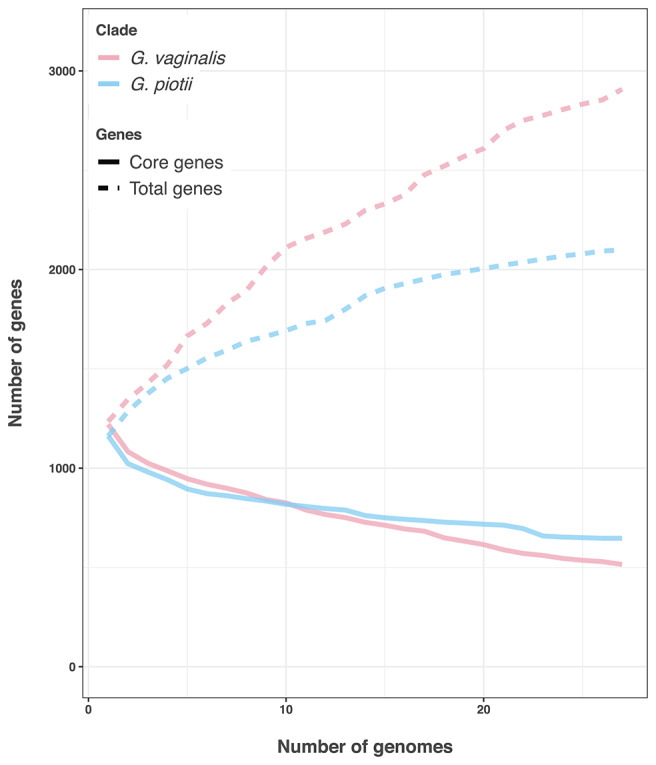
*G. vaginalis* has a larger accessory genome than *G. piotii*. Rarefaction curves of core and total gene content. *G. vaginalis* isolates were subsampled to the number of *G. piotii* isolates, and both species were iteratively sampled 100 times. The median value is shown. *G. vaginalis* has a larger accessory genome than *G. piotii*, consistent with acquisition of novel gene content from a diverse pool.

Differences in the amount of recombination between *G. vaginalis* and *G. piotii* appear to affect not only the core genome, but also the accessory genome. *G. vaginalis* has a larger pangenome than *G. piotii*, consistent with acquisition of novel gene content from diverse sources (Figure 12).

## Discussion

### Structure of *Gardnerella* spp. Pangenome

*Gardnerella* spp. comprise diverse bacteria with a distinct genetic structure (Ahmed et al., 2012). This diversity has been delineated both phylogenetically and metabolically (Ahmed et al., 2012; Cornejo et al., 2018; Vaneechoutte et al., 2019). Using WGS data from 106 *Gardnerella* isolates, we identified eight major clades from a core genome alignment that are consistent with published genomic analyses (Ahmed et al., 2012; Devault et al., 2017; Schellenberg et al., 2017; Cornejo et al., 2018; Vaneechoutte et al., 2019). Our data primarily consist of isolates from *G. vaginalis* and *G. piotii*, which are found most commonly in clinical samples with higher Nugent scores (Janulaitiene et al., 2017).

The eight major clades in our sample were differentiated with respect to both allelic variation in their core genomes (Figure 1) and gene content in their accessory genomes (Figure 2). Prior research found evidence of frequent homologous recombination within clades/species (Ahmed et al., 2012) and functional differentiation of the accessory genome (Cornejo et al., 2018). Our results extend these observations with the observation that allelic variants in the core genome appear to be more readily exchanged within species than between them (Figures 3, 8). In addition, we found evidence of barriers to recombination in the accessory genome, as shared accessory genes are more similar within species than between species (Figure 11). Accessory genes are not maintained at similar frequencies across species, suggesting that selection pressures for the shared accessory genes are not the same across species (Figure 10).

### Mechanisms and Barriers to LGT in *Gardnerella*

Previous studies identified competence genes in a handful of *Gardnerella* isolates (Yeoman et al., 2010). We systematically examined our sample for previously identified competence genes as well as genes known to encode tad pili (Tomich et al., 2007; Yeoman et al., 2010). We found these genes to be encoded by most isolates from the eight clades/species in our sample (Figure 4). The competence machinery, was however, highly genetically differentiated among clades/species (Figure S2). This raises the possibility of functional differentiation among competence genes, which could contribute to genetic isolation of clades/species (Porse et al., 2018). We investigated this hypothesis by computing the ratio of non-synonymous to synonymous variation in pairwise comparisons and across phylogenies of competence genes (Figure 5; Figure S3). We did not find any evidence of functional divergence among competence genes. Our results instead suggest that competence genes are under similar evolutionary pressures as the core genome, with both evolving under purifying selection (Figure 5; Figure S3).

A previous study identified predicted prophage genes in *Gardnerella* spp. (Malki et al., 2016). This does not necessarily indicate the presence of prophage, particularly if phage orthologs are not found within a cluster of phage associated genes. We used ProphET (Reis-Cunha et al., 2019) to identify prophage clusters, which we found in 70% of isolates (Figure 6). One limitation of this method is that prophage found in poorly assembled regions or across multiple contigs in the assembly may remain unidentified thus the true carriage frequency of prophage could be higher than our estimate. However, we blasted the prophage regions against the *de novo* assembled contigs and found that hits along the end of contigs are very small in length, suggesting they are not unidentified prophage (Figure S7).

CRISPR/*cas* are adaptive immune systems that can protect bacterial genomes from mobile genetic elements, such as phage, and thus could potentially shape and reinforce the genetic barriers in *Gardnerella* spp. Loss of CRISPR has been shown to enable the proliferation of mobile elements in *Enterococcus* (Pleckaityte et al., 2012; Hullahalli et al., 2018). We did not find an association between the presence of CRISPR/*cas* genes and the absence of any prophage clusters, suggesting these interactions may not be straightforward (Figure 6). Restriction modification (RM) is another mechanism by which foreign DNA is cleaved and thus prevented from integrating into bacterial genomes (Tock and Dryden, 2005). We found a wide diversity of RM genes at varying frequencies among *Gardnerella* spp. (Figure S9). The role they play in shaping and maintaining LGT barriers is unknown.

Differences in codon usage are another possible barrier to recombination among species (Tuller et al., 2011). To investigate this possibility, we compared codon usage between the two most well-sampled species. We did not, however, find evidence of differential codon usage among *G. vaginalis* and *G. piotii* isolates (Figure S10). It's intriguing that codon usage appears to be harmonized amongst these highly genetically differentiated species. By comparison, a study of a single relatively clonal species (*S. aureus*) found evidence of differences in codon usage among ecotypes (Richardson et al., 2018). We and others have found evidence that the pore-forming toxin vaginolysin is freely exchanged among *Gardnerella* spp. (Figure S5) (Ahmed et al., 2012). Harmonization of codon usage could facilitate exchange of genes like vaginolysin that are critical to diverse *Gardnerella* spp. We found evidence that vaginolysin genes are evolving under strong constraint (Figure 5A), which supports the idea that the toxin is important to the fitness of diverse *Gardnerella* spp.

Our results indicate that clades/species of *Gardnerella* spp. are reproductively isolated despite being found in the same niche. This differentiation does not appear to be driven by functional differentiation of competence genes, nor by differences in patterns of codon usage. The likelihood of LGT events and compatibility of transferred genes with the recipient genome increases between closely related genomes (Popa and Dagan, 2011). It is probable that general patterns of differentiation in the core and accessory genomes of *Gardnerella* spp. have a role maintaining species separation (Porse et al., 2018). It is intriguing that between-species LGT appears to have been more common in the remote past (Figure 3B), when the species may have shared more sequence similarity. Other potential mediators of reproductive isolation among species may be restriction-modification systems and CRISPR/*cas* systems, both of which can target degradation of foreign DNA, and thus shape patterns of LGT (Tock and Dryden, 2005; Marraffini and Sontheimer, 2008; Dupuis et al., 2013; Hullahalli et al., 2018). We did not find evidence of interactions between CRISPR/*cas* loci and prophage (Figure 6); suggesting interactions among these elements are complex in *Gardnerella* spp.

### Patterns of Within and Between Species LGT in *G. vaginalis* and *G. piotii*

We found patterns of LGT to vary among *Gardnerella* spp. Specifically, we found evidence of distinct patterns of within-species recombination in comparisons of *G. vaginalis* and *G. piotii*. *G. vaginalis* appears to engage more frequently in LGT (Figure 8; Figure S11) as a larger proportion of each isolate's core genome is estimated to have been affected by recombination (Figure 9). Additionally, *G. vaginalis* has a larger pangenome (Figure 12), consistent with higher levels of gene importation in the accessory genome. These findings raise the possibility that *G. vaginalis* exchanges DNA with more diverse partners than does *G. piotii*, potentially with other non-*Gardnerella* members of the complex polymicrobial BV biofilm.

We do not know the underlying mechanism for differences in LGT between *G. vaginalis* and *G. piotii*. Variation in rates of transduction as well as restriction-modification systems could play a role. A previous study found *G. vaginalis* to be enriched for “phage-associated protein” and three genes involved in a type I RM system (Cornejo et al., 2018). We observed a wide diversity of RM systems, with multiple type I variants found within each species (Figure S9). Thus, even if RM systems explain the differences in LGT patterns within species, it is not a one to one relationship between species and RM systems. Teasing apart those interactions will require more research.

In conclusion, *Gardnerella* spp. are genetically distinct in both their core and accessory genomes. We found evidence of more within species LGT in the core and accessory genomes, suggesting active maintenance of reproductive barriers despite similar patterns of codon usage. The putative competence machinery is genetically differentiated between clades/species; however, we found no evidence of functional divergence/positive selection driving clade/species separation. We identified a larger pangenome in *G. vaginalis* than in *G. piotii* as well as more LGT in the core genome, suggesting *G. vaginalis* engages more frequently in LGT with more diverse partners. Taken together, our results demonstrate that co-localized bacterial populations can maintain a complex genetic structure in which genetic exchange appears to be restricted to specific sub-populations with exceptions for individual genes (e.g., vaginolysin). The forces maintaining this structure are yet to be fully elucidated but likely include patterns of sequence similarity and possibly phage, CRISPR/*cas*, RM systems and interactions among them. Defining evolutionary interactions in bacterial populations helps to illuminate how clinically important traits such as antibiotic resistance and virulence emerge and are maintained in these complex communities.

## Data Availability Statement

The raw data supporting the conclusions of this article are freely available at NCBI, PRJNA602880.

## Author Contributions

CP and TM conceived of the study. CP developed the study design, with input from the other authors. LB and TM performed the analyses. LB drafted the manuscript with input from CP. All authors provided critical feedback and contributed to manuscript revision.

## Conflict of Interest

The authors declare that the research was conducted in the absence of any commercial or financial relationships that could be construed as a potential conflict of interest.
