# Supplementary material for: Lateral Gene Transfer Shapes Diversity of Gardnerella spp
Source: Front Cell Infect Microbiol. 2020 Jun 23;10:293. doi: 10.3389/fcimb.2020.00293 (PMC7324480; doi:10.3389/fcimb.2020.00293)
Supplement: Supplementary file 1 [file Data_Sheet_1.PDF]

Table S1. Accessions for *Gardnerella* spp. isolates sequenced in this study.

Strain	Accession	Location
GV1	SRR10957307	Madison, WI, USA
GV24	SRR10957311	Australia
GV25	SRR10957310	Australia
GV26	SRR10957309	Australia
GV28	SRR10957308	Australia
GVAR737	SRR10957315	New York, USA
GVAR738	SRR10957314	New York, USA
GVAR739	SRR10957313	New York, USA
GVAR741	SRR10957312	New York, USA

Table S2. Strain accession numbers and available clinical data for all isolates in this study.

Strain	Accession	Source	Symptoms
101	GCA_000165615.2	-	-
40905	GCA_000025205.1	vagina	BV+
55152	GCA_000263475.1	vagina	BV+
75712	GCA_000263535.1	vagina	BV-
00703B	GCA_000263615.1	vagina	BV+
00703C2	GCA_000263515.1	vagina	BV+
00703D	GCA_000263635.1	vagina	BV-
0288E	GCA_000263555.1	endometrium	BV+
1400E	GCA_000263495.1	endometrium	BV+
14019_MetR	GCA_001278345.1	lab culture of 14019	BV-
1500E	GCA_000263595.1	endometrium	BV+
18_4	GCA_001660755.1	urine	-
23_12	GCA_001660735.1	urine	-
284V	GCA_000263435.1	vagina	BV+
315A	GCA_000214315.2	vagina	unknown
41V	GCA_000165635.2	vagina	BV-
5_1	GCA_000176495.1	vagina	BV-
6119V5	GCA_000263655.1	vagina	BV-
6420B	GCA_000263575.1	vagina	None

AMD	GCA_000176475.1	vagina	BV+
ATCC_49145	GCA_003034925.1	vagina	BV+
ATCC14018	GCA_003397685.1	vagina	BV-
ATCC14019	GCA_000159155.2	vagina	BV-
CMW7778B	GCA_001563665.1	vagina	-
DNF01149	GCA_002894105.1	vagina	-
DSM_4944	GCA_900105405.1	-	-
FDAARGOS_296	GCA_002206225.2	-	-
FDAARGOS_568	GCA_003812765.1	-	-
GED7275B	GCA_001546445.1	vagina	-
GED7760B	GCA_001546455.1	vagina	-
GH007	GCA_003426405.1	vaginal fluid	-
GH015	GCA_003408745.1	vaginal fluid	-
GH019	GCA_003426565.1	vaginal fluid	-
GH020	GCA_003426385.1	vaginal fluid	-
GH021	GCA_003426345.1	vaginal fluid	-
GS_10234	GCA_003397745.1	vagina	-
GS_9838_1	GCA_003397705.1	vagina	-
GV1	SRR10957307	-	-
GV24	SRR10957311	-	-
GV25	SRR10957310	-	-
GV26	SRR10957309	-	-
GV28	SRR10957308	-	-
Gv3549624	GCA_001049785.1	vagina	unknown
GV37	GCA_001953155.1	blood culture	bacteremia associated with severe acute encephalopathy
GVAR737	SRR10957315	-	-
GVAR738	SRR10957314	-	-
GVAR739	SRR10957313	-	-
GVAR741	SRR10957312	-	-
HMP9231	GCA_000213955.1	vagina	BV-
JCP7275	GCA_000414705.1	vagina	BV+
JCP7276	GCA_000414685.1	vagina	BV intermediate
JCP7659	GCA_000414665.1	vagina	BV+
JCP7672	GCA_000414645.1	vagina	BV-
JCP7719	GCA_000414625.1	vagina	BV+
JCP8017A	GCA_000414605.1	vagina	BV+

JCP8017B	GCA_000414585.1	vagina	BV+
JCP8066	GCA_000414565.1	vagina	BV-
JCP8070	GCA_000414545.1	vagina	BV+
JCP8108	GCA_000414525.1	vagina	BV+
JCP8151A	GCA_000414505.1	vagina	BV+
JCP8151B	GCA_000414485.1	vagina	BV+
JCP8481A	GCA_000414465.1	vagina	BV+
JCP8481B	GCA_000414445.1	vagina	BV+
JCP8522	GCA_000414425.1	vagina	BV+
KA00225	GCA_002896555.1	vagina	-
N101	GCA_003369895.1	vagina	-
N144	GCA_003408835.1	vagina	-
N153	GCA_003369935.1	vagina	-
N160	GCA_003408775.1	vagina	-
N165	GCA_003408785.1	vagina	-
N72	GCA_003408815.1	vagina	-
N95	GCA_003369965.1	vagina	-
NR001	GCA_003426545.1	vaginal fluid	-
NR010	GCA_003408845.1	vaginal fluid	-
NR037	GCA_003426445.1	vaginal fluid	-
NR038	GCA_003585655.1	vaginal fluid	-
NR039	GCA_003585755.1	vaginal fluid	-
PSS_7772B	GCA_001546485.1	urine	-
troy	PRJNA352376	-	-
UGent_06_41	GCA_003293675.1	vagina	-
UGent_09_01	GCA_003397755.1	vagina	-
UGent_09_07	GCA_003397665.1	vagina	-
UGent_09_48	GCA_003397635.1	vagina	-
UGent_18_01	GCA_003397585.1	vagina	-
UGent_21_28	GCA_003397615.1	vagina	-
UGent_25_49	GCA_003397605.1	vagina	-
UMB0032A	GCA_002862015.1	catheter	-
UMB0032B	GCA_002862005.1	catheter	-
UMB0061	GCA_002861165.1	catheter	-
UMB0170	GCA_002884855.1	catheter	-
UMB0233	GCA_002862045.1	catheter	-
UMB0264	GCA_002884875.1	catheter	-

UMB0298	GCA_002861975.1	catheter	-
UMB0386	GCA_002861965.1	catheter	-
UMB0682	GCA_002862065.1	catheter	-
UMB0768	GCA_002884835.1	catheter	-
UMB0770	GCA_002861945.1	catheter	-
UMB0775	GCA_002861925.1	catheter	-
UMB0830	GCA_002861905.1	catheter	-
UMB0833	GCA_002861885.1	catheter	-
UMB0912	GCA_002861125.1	catheter	-
UMB0913	GCA_002861145.1	catheter	-
UMB1642	GCA_002884795.1	catheter	-
UMB1686	GCA_002884775.1	catheter	-
W11	GCA_003369875.1	vagina	-
WP023	GCA_003426285.1	vaginal fluid	-

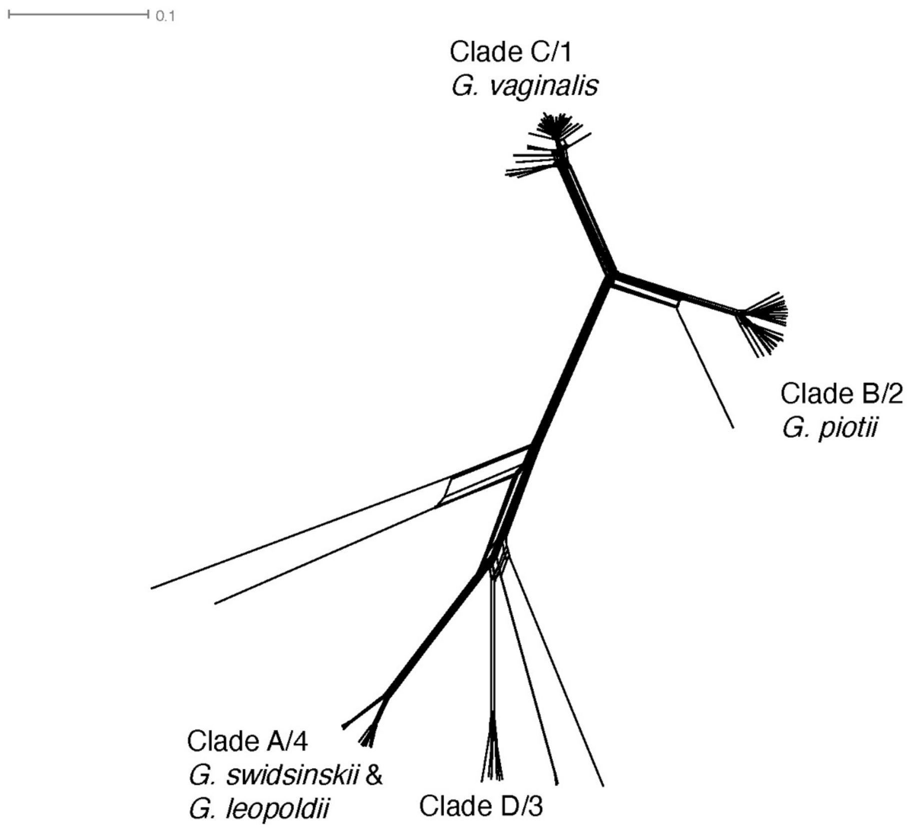

Figure S1. Core genome phylogenetic network contains deeply branched lineages corresponding to *Gardnerella* spp. Core genome phylogenetic network of *Gardnerella* spp. Reticulations indicate uncertainty in placement of that isolate (tip) in the network, which can be the result of recombination. A long branch separates *G. vaginalis* and *G. piotii* from the other clades/species. Some deeply branched lineages are represented by single isolates, whereas the majority of the sample clusters in *G. vaginalis, G. piotii, G. swidskinski,* and *G. leopoldii*.

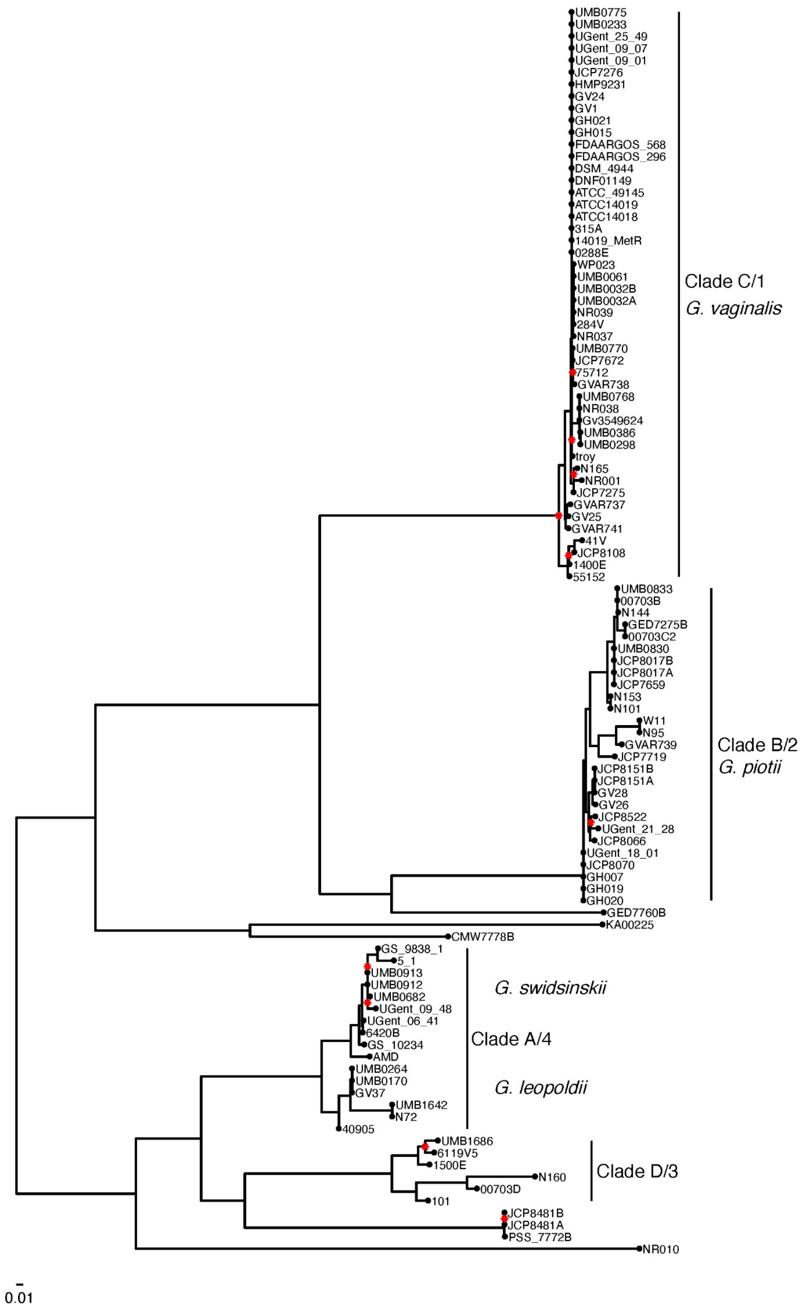

Figure S2. Homologs of *comEA* have diversified along with the core genome. Homologs of *comEA* were found in 104 out of 106 total isolates. A maximum likelihood phylogeny of *comEA* was estimated using FastTree. The phylogeny is midpoint rooted, and nodes with bootstrap values lower than 70 shown in red. Branch lengths are scaled by the number of substitutions per site. The clade structure of the *comEA* tree is similar to that of the core genome phylogeny.

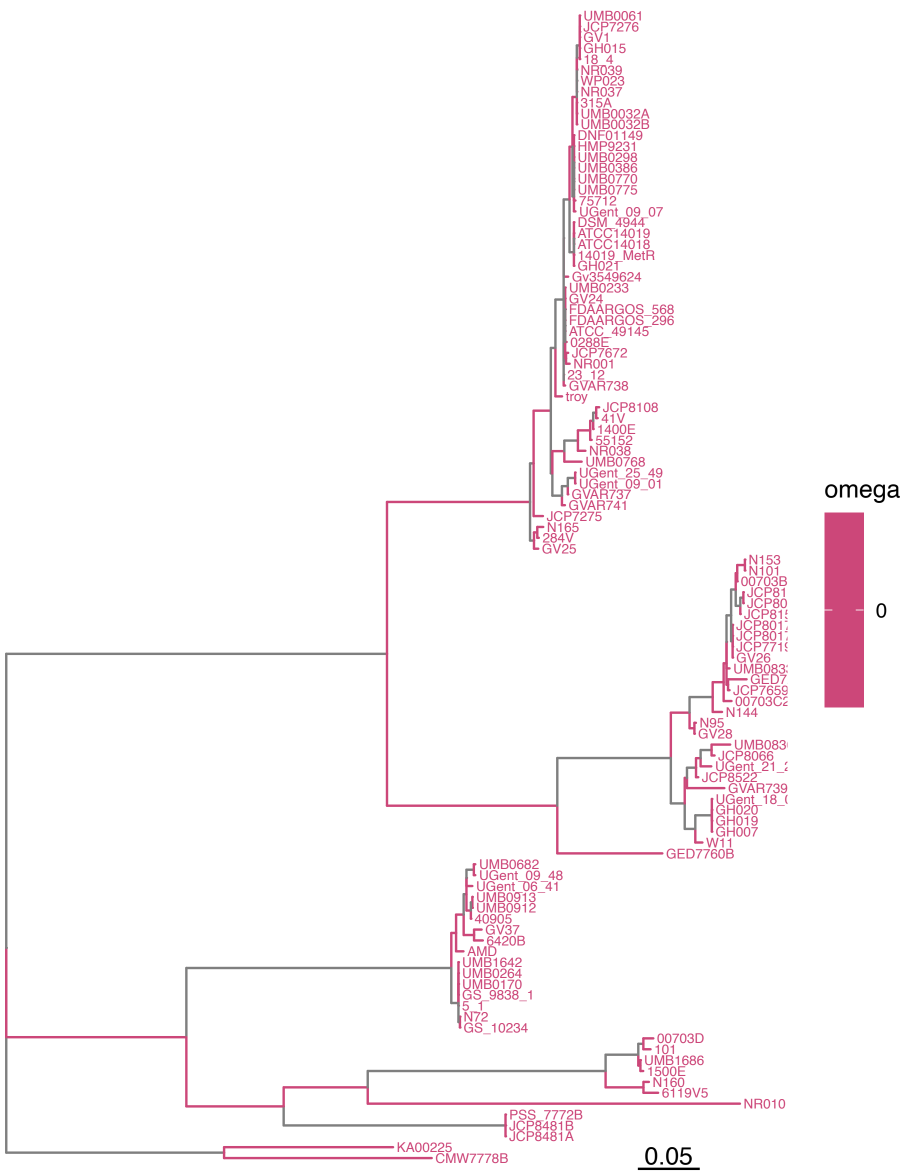

(A)

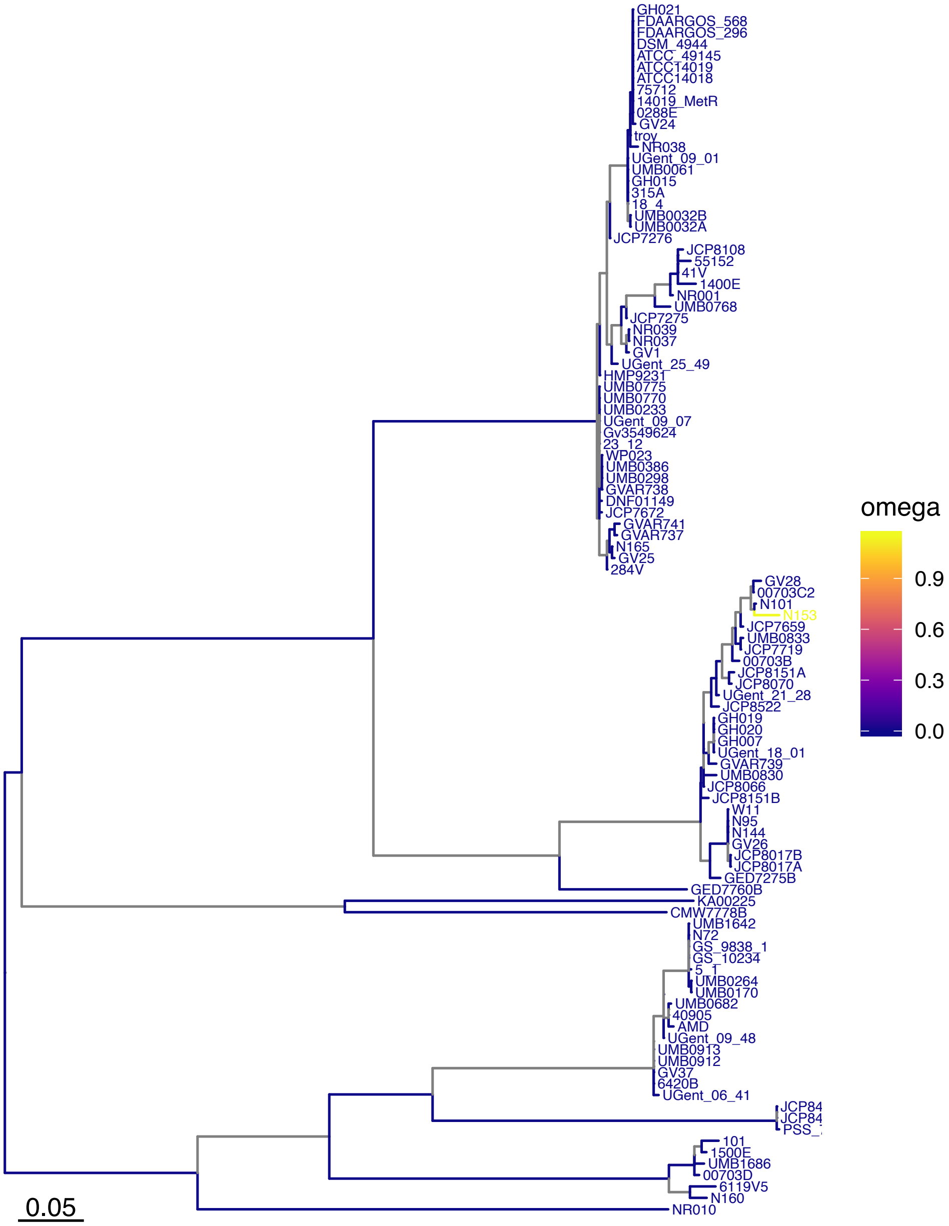
GH021
FDAARGOS_568
FDAARGOS_296
DSM_4944
ATCC_49145
ATCC14019
ATCC14018
75712
14019_MetR
0288E
GV24
troy
NR038
UGent_09_01
UMB0061
GH015
315A
18_4
UMB0032B
UMB0032A
JCP7276
JCP8108
55152
41V
1400E
NR001
UMB0768
JCP7275
NR039
NR037
GV1
UGent_25_49
HMP9231
UMB0775
UMB0770
UMB0233
UGent_09_07
Gv3549624
23_12
WP023
UMB0386
UMB0298
GVAR738
DNF01149
JCP7672
GVAR741
GVAR737
N165
GV25
284V
GV28
00703C2
N101
N153
JCP7659
UMB0833
JCP7719
00703B
JCP8151A
JCP8070
UGent_21_28
JCP8522
GH019
GH020
GH007
UGent_18_01
GVAR739
UMB0830
JCP8066
JCP8151B
W11
N95
N144
GV26
JCP8017B
JCP8017A
GED7275B
GED7760B
KA00225
CMW7778B
UMB1642
N72
GS_9838_1
GS_10234
5_1
UMB0264
UMB0170
UMB0682
40905
AMD
UGent_09_48
UMB0913
UMB0912
GV37
6420B
UGent_06_41
101
1500E
UMB1686
00703D
6119V5
N160
NR010
0.05
omega
0.9
0.6
0.3
0.0

(B)

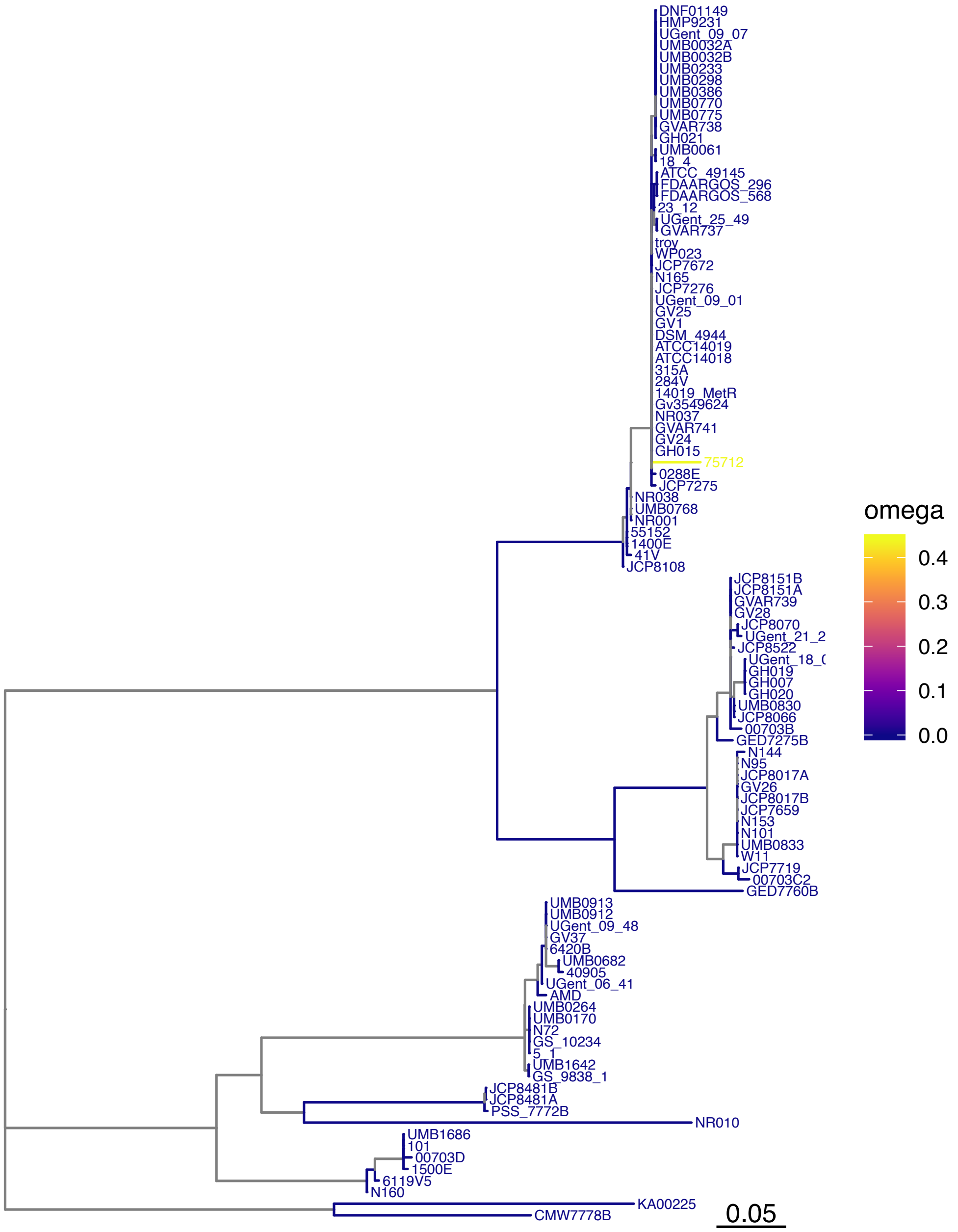

(C)

Figure S3. Purifying selection is the primary force shaping diversity of competence genes in *Gardnerella* spp. Proportion of sites under positive selection along branches of maximum likelihood gene phylogenies of (A) *cpaF* (B) *tadC* (C) *tadG*. Using the aBSREL test in HyPhy, we identified branches with significant evidence ($p < 0.05$) of selection. Branch specific omega values show little evidence of positive selection, suggesting purifying selection.

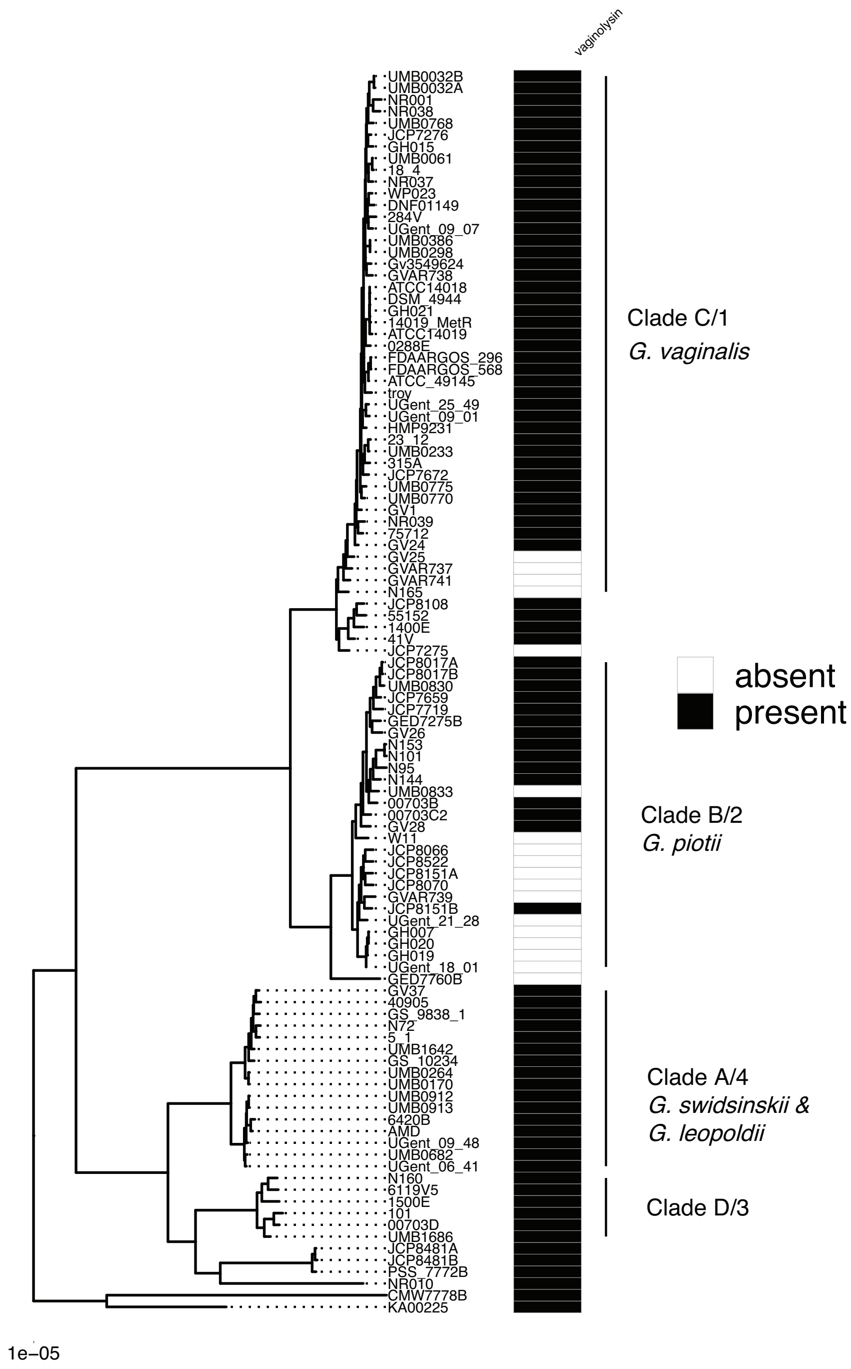

Figure S4. Vaginolysin is an accessory gene of *Gardnerella* spp. Presence absence matrix of vaginolysin across *Gardnerella* spp. ordered according to the core genome phylogeny (left). Vaginolysin is found in 83% of isolates. The presence absence matrix implies that vaginolysin has been gained and during the evolution of our sample.

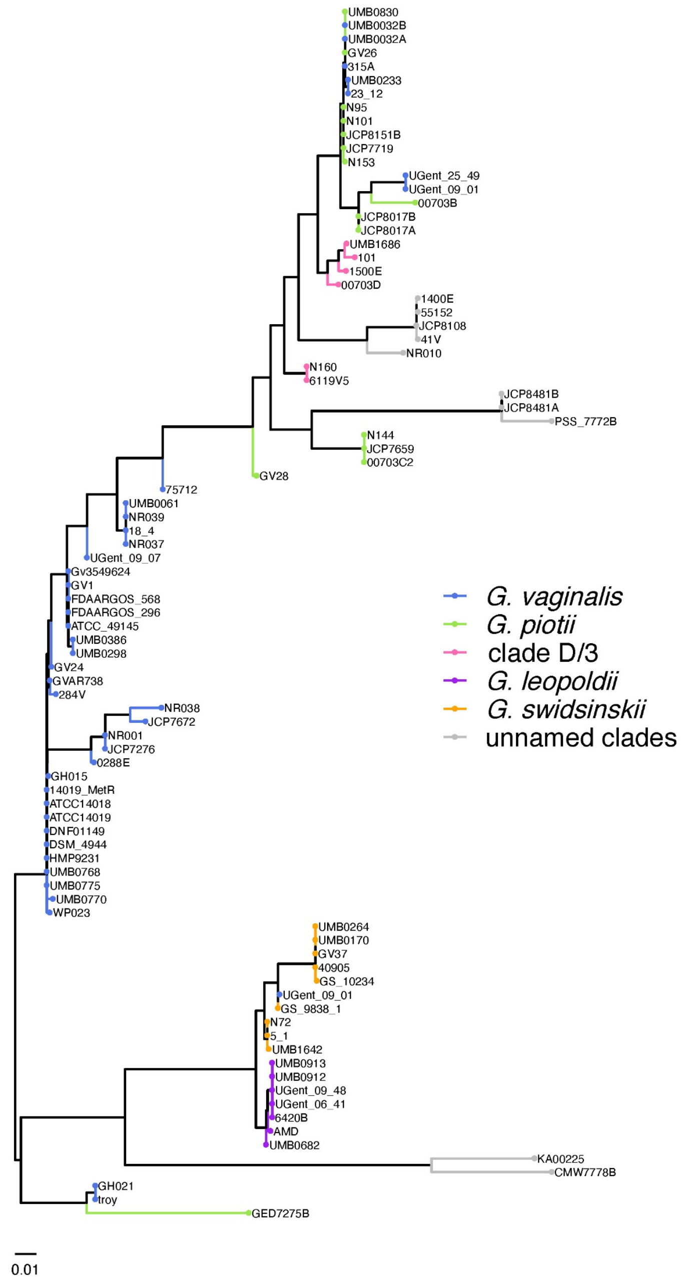

Figure S5. Maximum likelihood phylogeny of vaginolysin gene does not mirror that of core genome phylogeny, suggesting between species recombination. Homologs of vaginolysin were found in 88 out of 106 total isolates at an amino acid similarity threshold of >80%. A maximum likelihood phylogeny of vaginolysin was estimated using FastTree. The phylogeny is midpoint rooted, and isolates are colored according to spp. designation. Branch lengths are scaled by the number of substitutions per site. The clade structure of the vaginolysin tree does not mirror that of the core genome phylogeny.

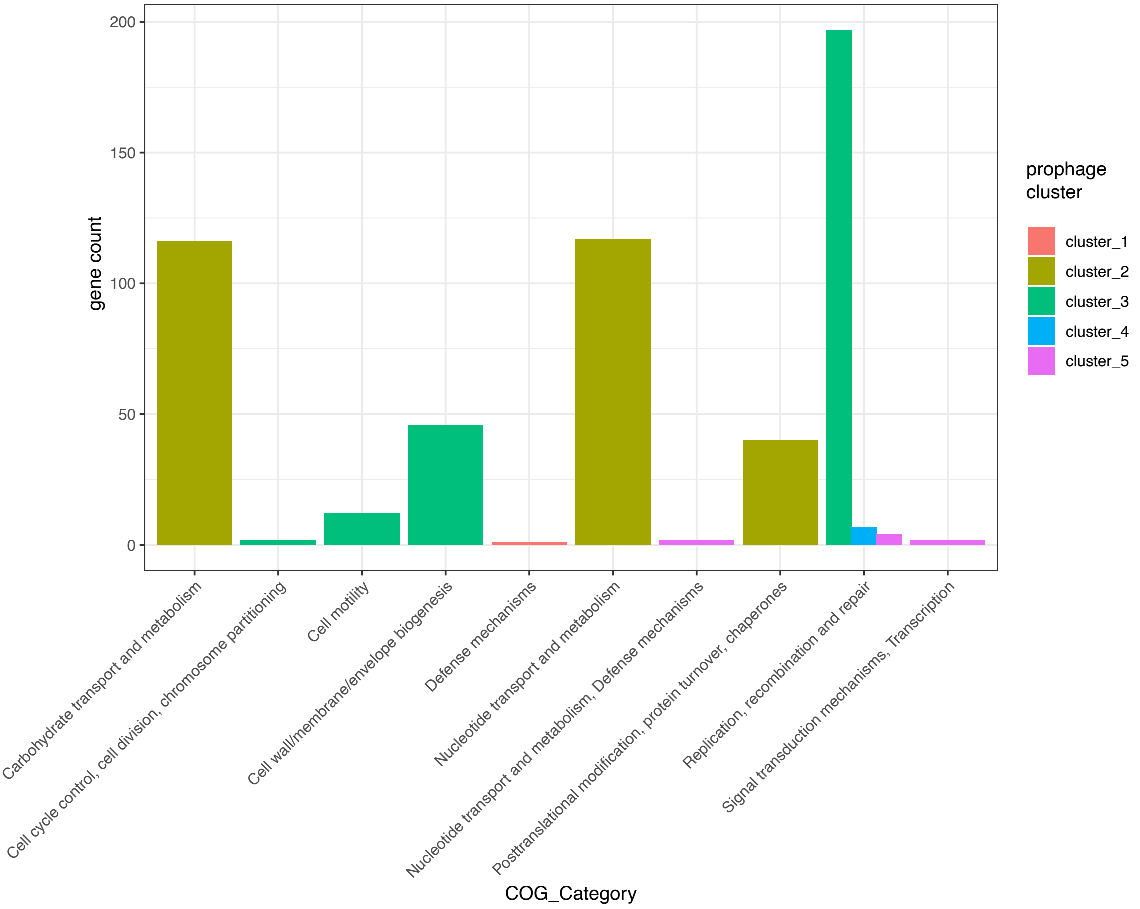

Figure S6. Gene content varies between prophage cluster. Number of genes found in Clusters of Orthologous Groups (COG) of phage sequences colored according to phage cluster. We calculated pairwise mash distances of the nucleotide sequences to define prophage clusters. The majority of annotated genes were uncharacterized hypothetical genes (82%).

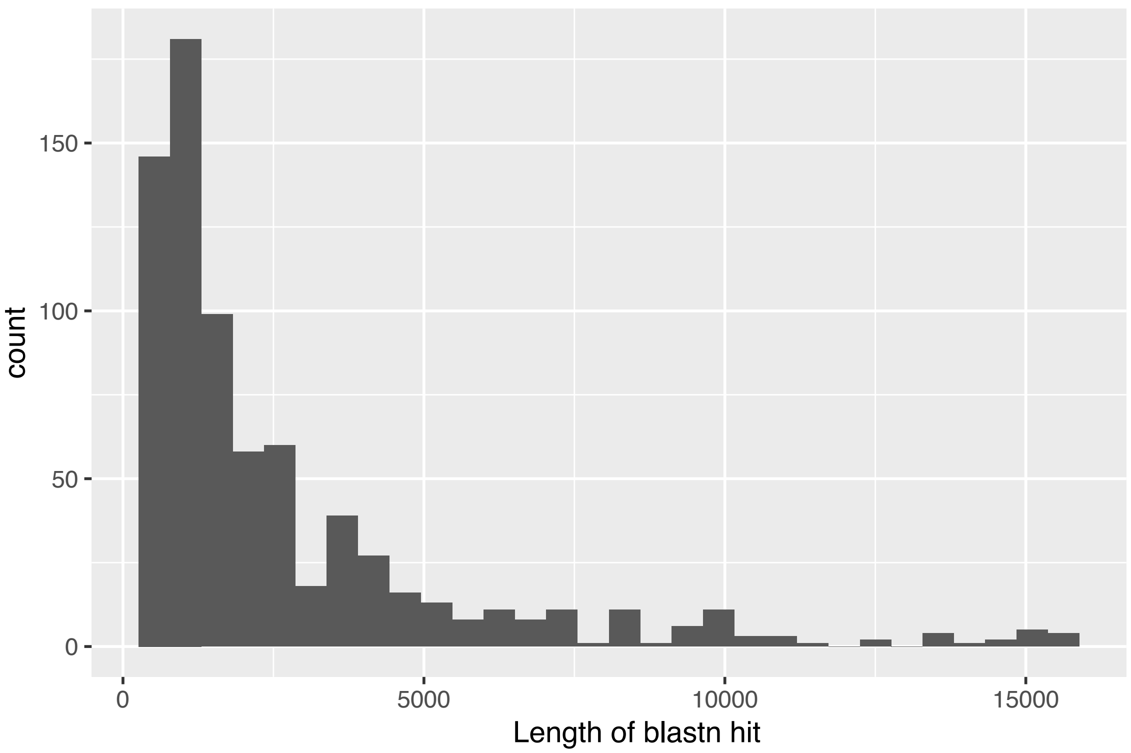

Figure S7. To look for prophage that had been split between *de novo* assembled contigs, and thus unidentified by ProphET, we created a custom nucleotide blast database using all of the assembled contigs from the 106 Gardnerella *spp.* isolates. We blasted the prophage regions identified by ProphET and filtered for hits found within 50 bp of the end of a contig. Of those hits found near the end of contigs, the majority are very small in length, which suggests they are not likely to be unidentified prophage.

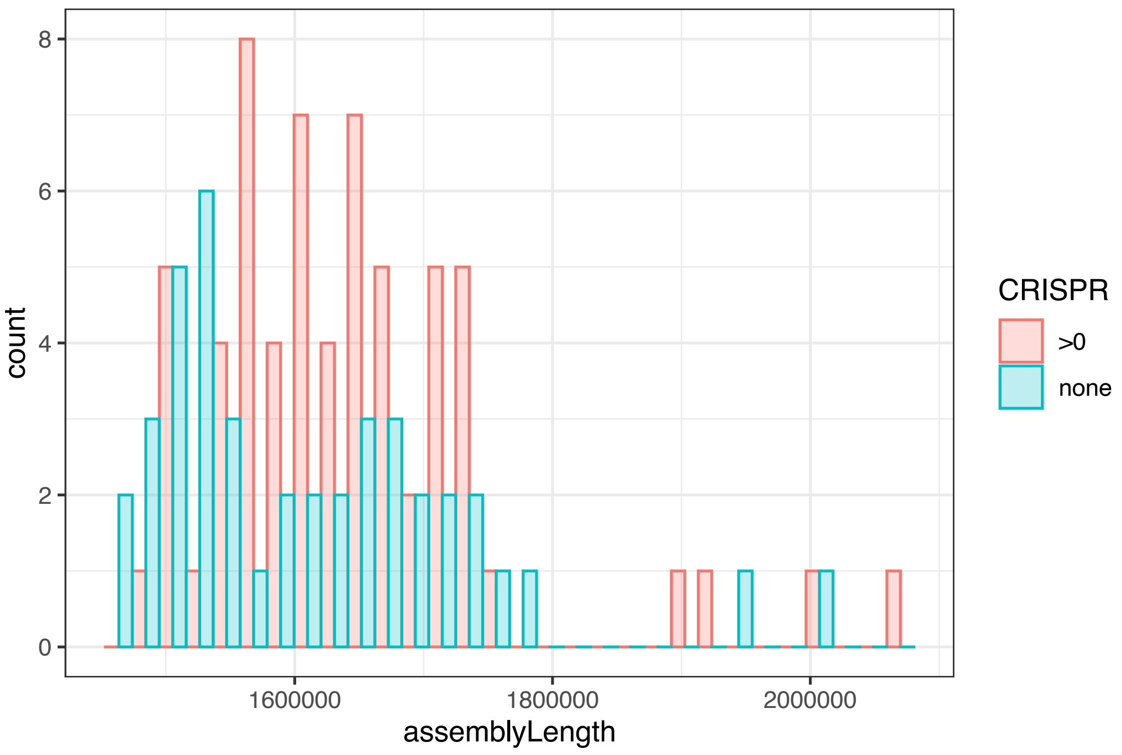

Figure S8. CRISPR/*cas* is not associated with genome length. Distribution of de novo assembly genome length of isolates with and without CRISPR/*cas* genes. Loss of CRISPR/*cas* can lead to proliferation of mobile genetic elements (Hullahalli et al., 2018), which may cause the total genome length to increase. To test this hypothesis, we compared the presence of CRISPR/*cas* to the total length of *de novo* assembled contigs. Genome length for isolates with and without CRISPR/*cas* genes were not significantly different (Mann-Whitney-Wilcoxon test, W = 1058, p = 0.08).

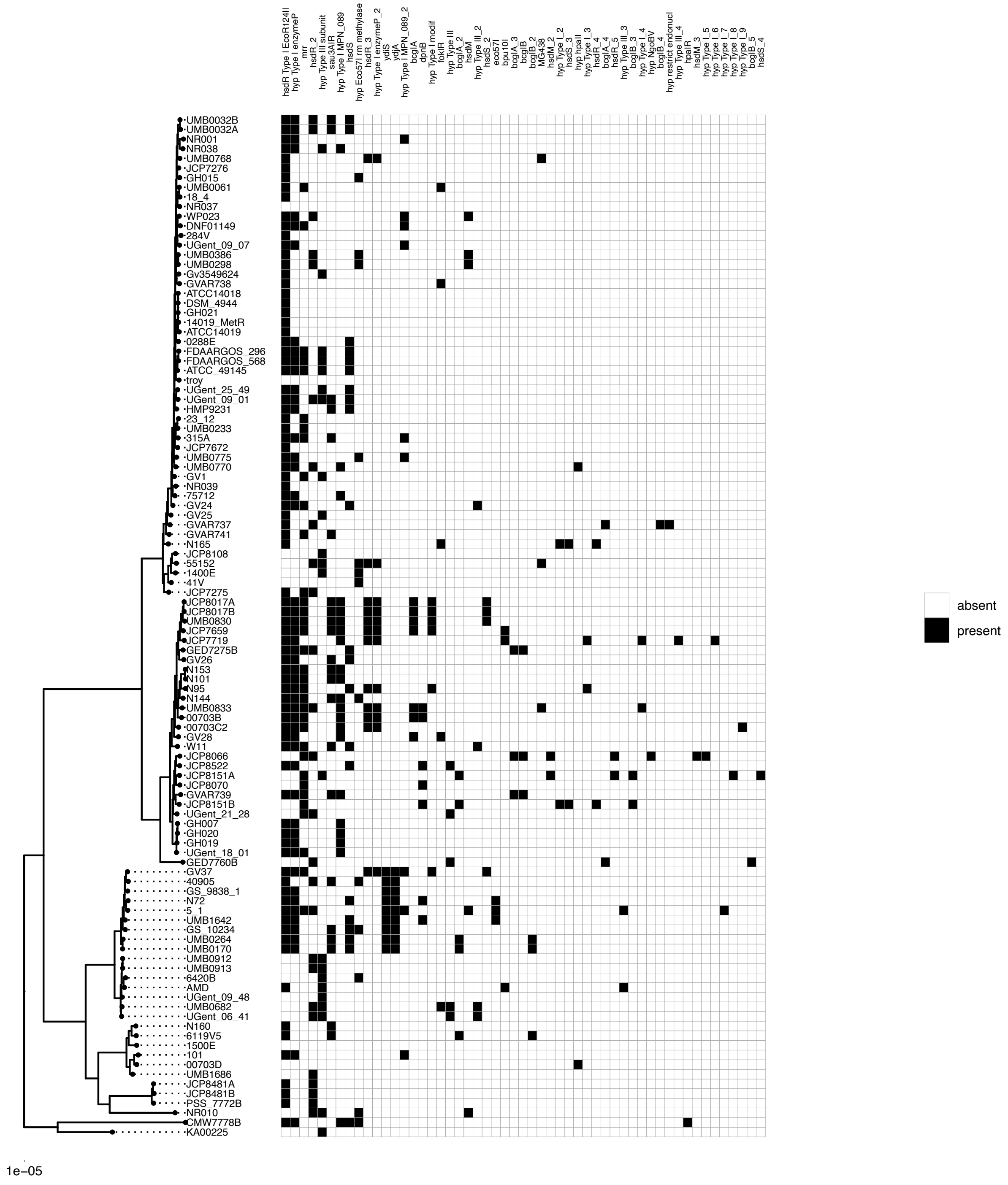

Figure S9. *Gardnerella* spp. contain a wide diversity of restriction modification genes. Core genome phylogeny of *Gardnerella* spp. isolates (left) with restriction modification presence absence matrix (right). We identified genes involved in restriction modification systems using PIRATE pangenome output and Prokka genome annotations.

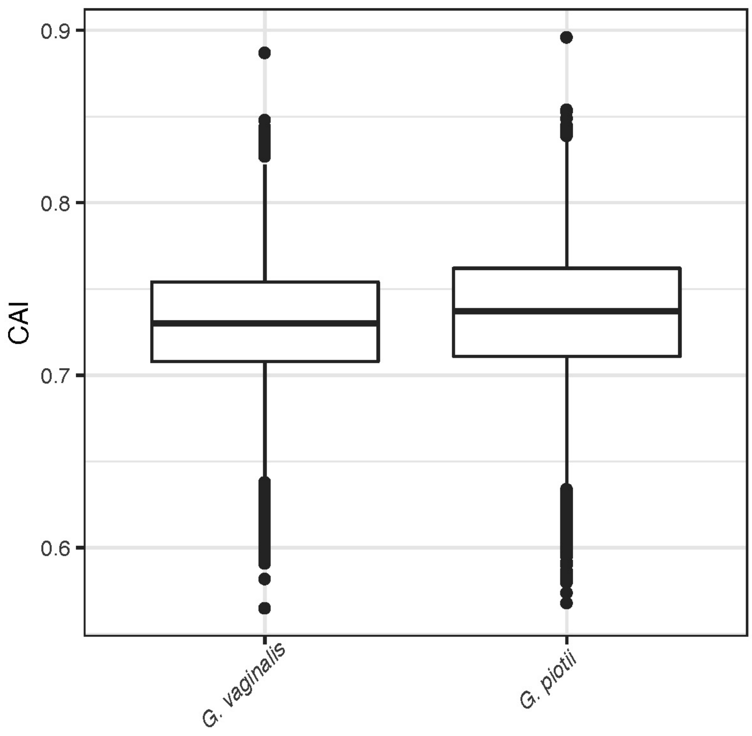

Figure S10. Codon usage in *G. vaginalis* is similar to that of *G. piotii.* Codon adaptation index (CAI) for core genes in *G. vaginalis* and *G. piotii*. The distributions are similar, indicating codon usage is not differentiated in the core genomes despite barriers to recombination between species (t-test, $p > 0.99$).

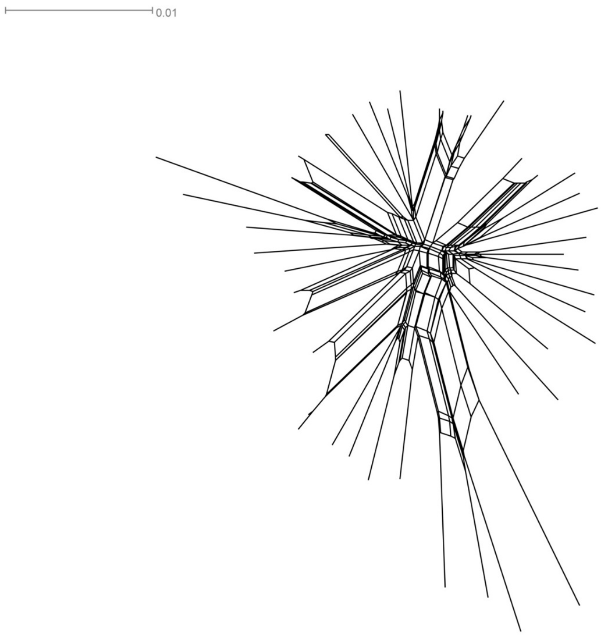

(A)

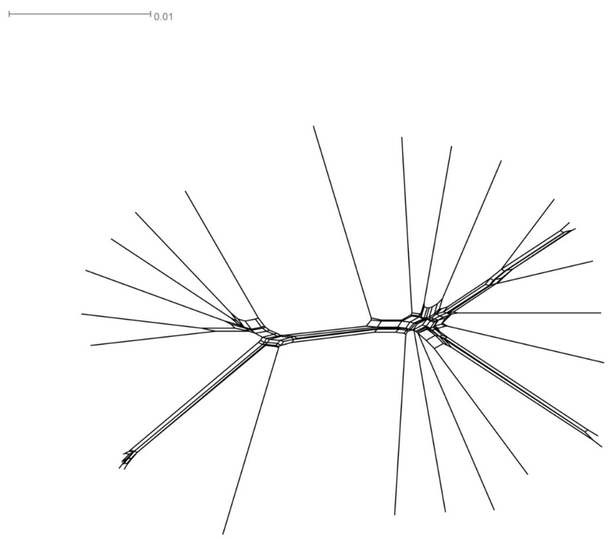

(B)

Figure S11. Phylogenetic networks of the core genomes of *G. vaginalis* (A) and *G. piotii* (B) show more evidence of recombination in *G. vaginalis* than *G. piotii.* Phylogenetic networks of *G. vaginalis* (A) and *G. piotii* (B) core genomes. The networks were estimated using SplitsTree4 (Huson and Bryant, 2005) with gapped regions masked. Both networks have a star like topology, consistent with rapid population expansion. More reticulations are evident in the *G. vaginalis* network, which is consistent with relatively higher levels of recombination.
